# Supplementary material for: SLITRK2 variants associated with neurodevelopmental disorders impair excitatory synaptic function and cognition in mice
Source: Nat Commun. 2022 Jul 15;13:4112. doi: 10.1038/s41467-022-31566-z (PMC9287327; doi:10.1038/s41467-022-31566-z)
Supplement: Supplementary file 1 — Supplementary Information [file 41467_2022_31566_MOESM1_ESM.pdf]

Supplementary Information for Chehadeh, Han, Kim, Jang *et al*., "SLITRK2 variants associated with neurodevelopmental disorders impair excitatory synaptic function and cognition in mice"

Supplementary Figures 1 to 20

Supplementary Tables 1, 2, 3 and 4

Supplementary Notes

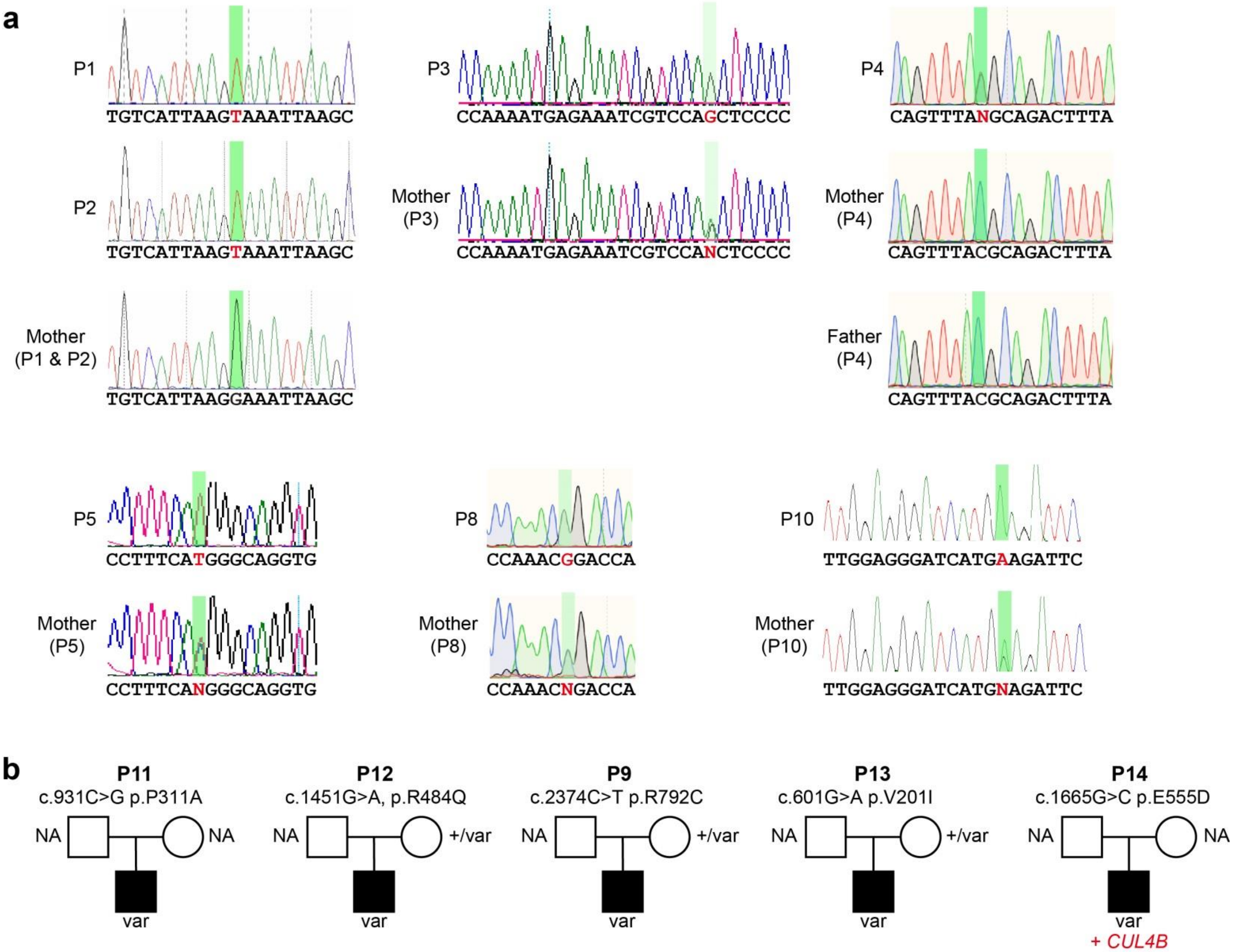

Supplementary Figure 1. Additional variants identified in *SLITRK2* in individuals with NDD.

a Sanger sequencing traces of P1, P2, P3, P4, P5, P8 and P10 with *SLITRK2* variants. Mutated residues are highlighted in red.

b Pedigrees of families with additional rare variants of *SLITRK2*. These variants were not considered to be probably disease-causing because all, except for the E555D variant, have been reported in males from the gnomAD population. The E555D variant was reported in an individual having another molecular diagnosis (a pathogenic variant in *CUL4B*) sufficient to explain his phenotype.

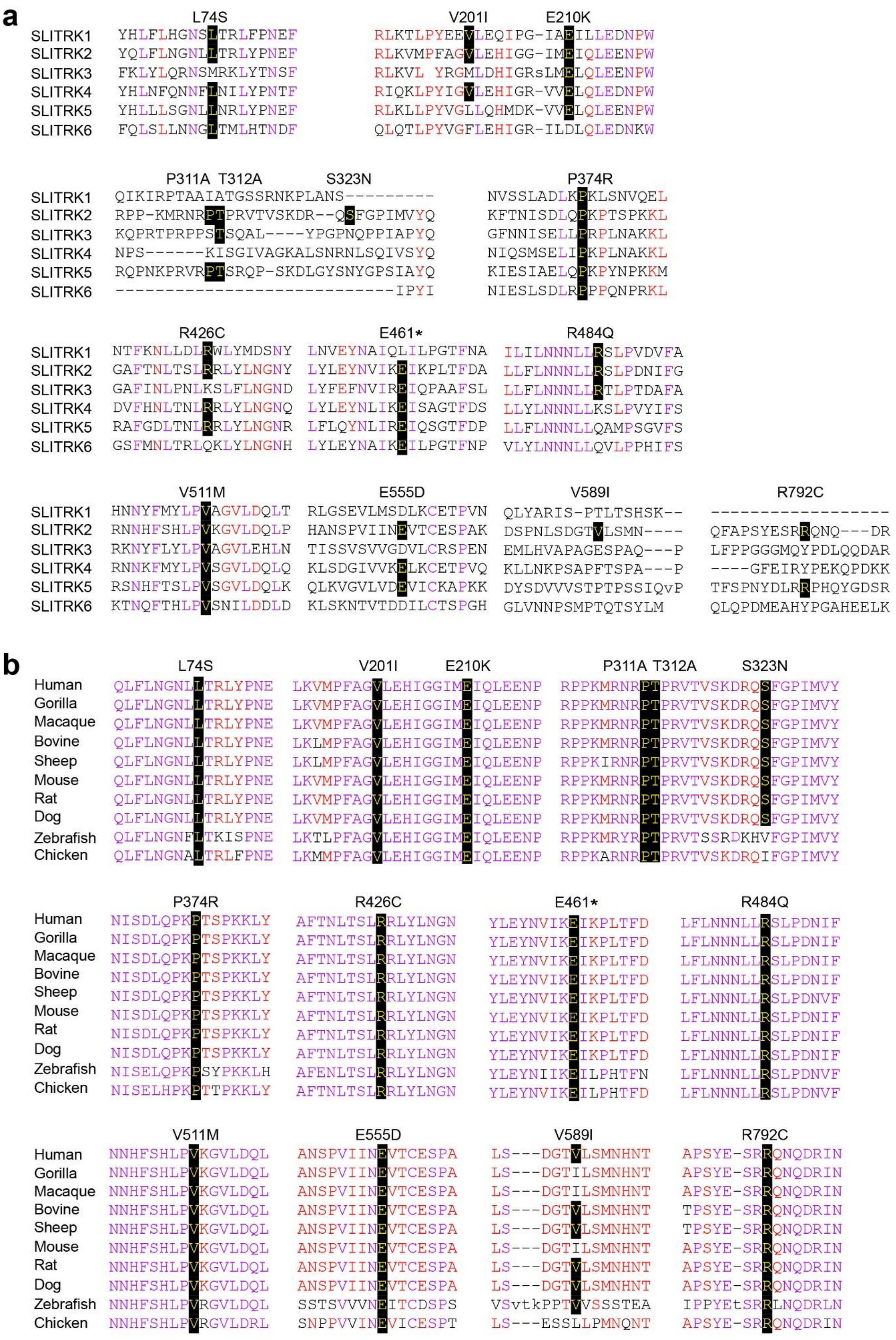
a
L74S
V201I
E210K
SLITRK1 YHLFLHGNSLTRLFPNEF RLKTLPYEEVLEQIPG-IAEILLEDNPW
SLITRK2 YQLFLNGNLLTRLYPNEF RLKVMPFAGVLEHIGG-IMEIQLEENPW
SLITRK3 FKLYLQRNSMRKLYTNSF RLKVL YRGMLDHIGRsLMELQLEENPW
SLITRK4 YHLNFQNNFLNILYPNTF RIQKLPYIGVLEHIGR-VVELQLEDNPW
SLITRK5 YHLLLSGNLLNRLYPNEF RLKLLPYVGLLQHMDK-VVELQLEENPW
SLITRK6 FQLSLLNNGLTMLHTNDF QLQTLPYVGFLEHIGR-ILDLQLEDNKW
P311A T312A
S323N
P374R
SLITRK1 QIKIRPTAAIATGSSRNKPLANS--------- NVSSLADLKPKLSNVQEL
SLITRK2 RPP-KMRNRPTPRVTVSKDR--QSFGPIMVYQ KFTNISDLQPKPTSPKKL
SLITRK3 KQPRTPRPPSTSQAL----YPGPNQPPIAPYQ GFNNISELLPRPLNAKKL
SLITRK4 NPS------KISGIVAGKALSNRNLSQIVSYQ NIQSMSELIPKPLNAKKL
SLITRK5 RQPNKPRVRPTSRQP-SKDLGYSNYGPSIAYQ KIESIAELQPKPYNPKKM
SLITRK6 ----------------------------IPYI NIESLSDLRPPPQNPRKL
R426C
E461*
R484Q
SLITRK1 NTFKNLLDLRWLYMDSNY LNVEYNAIQLILPGTFNA ILILNNNLLRSLPVDVFA
SLITRK2 GAFTNLTSLRRLYLNGNY LYLEYNVIKEIKPLTFDA LLFLNNNLLRSLPDNIFG
SLITRK3 GAFINLPNLKSLFLNGND LYFEFNVIREIQPAAFSL LLFLNNNLLRTLPTDAFA
SLITRK4 DVFHNLTNLRRLYLNGNQ LYLEYNLIKEISAGTFDS LLYLNNNLLKSLPVYIFS
SLITRK5 RAFGDLTNLRRLYLNGNR LFLQYNLIREIQSGTFDP LLFLNNNLLQAMPSGVFS
SLITRK6 GSFMNLTRLQKLYLNGNH LYLEYNAIKEILPGTFNP VLYLNNNLLQVLPPHIFS
V511M
E555D
V589I
R792C
SLITRK1 HNNYFMYLPVAGVLDQLT RLGSEVLMSDLKCETPVN QLYARIS-PTLTSHSK-- ------------------
SLITRK2 RNNHFSHLPVKGVLDQLP HANSPVIINEVTCESPAK DSPNLSDGTVLSMN---- QFAPSYESRRQNQ---DR
SLITRK3 RKNYFLYLPVAGVLEHLN TISSVSVVGDVLCRSPEN EMLHVAPAGESPAQ---P LFPPGGGMQYPDLQQDAR
SLITRK4 RNNKFMYLPVSGVLDQLQ KLSDGIVVKELKCETPVQ KLLNKPSAPFTSPA---P ----GFEIRYPEKQPDKK
SLITRK5 RSNHFTSLPVSGVLDQLK QLKVGVLVDEVICKAPKK DYSDVVVSTPTPSSIQvP TFSPNYDLRRPHQYGDSR
SLITRK6 KTNQFTHLPVSNILDDLD KLSKNTVTDDILCTSPGH GLVNNPSMPTQTSYLM QLQPDMEAHYPGAHEELK
b
L74S
V201I
E210K
P311A T312A
S323N
Human QLFLNGNLLTRLYPNE LKVMPFAGVLEHIGGIMEIQLEENP RPPKMRNRPTPRVTVSKDRQSFGPIMVY
Gorilla QLFLNGNLLTRLYPNE LKVMPFAGVLEHIGGIMEIQLEENP RPPKMRNRPTPRVTVSKDRQSFGPIMVY
Macaque QLFLNGNLLTRLYPNE LKVMPFAGVLEHIGGIMEIQLEENP RPPKMRNRPTPRVTVSKDRQSFGPIMVY
Bovine QLFLNGNLLTRLYPNE LKLMPFAGVLEHIGGIMEIQLEENP RPPKMRNRPTPRVTVSKDRQSFGPIMVY
Sheep QLFLNGNLLTRLYPNE LKLMPFAGVLEHIGGIMEIQLEENP RPPKIRNRPTPRVTVSKDRQSFGPIMVY
Mouse QLFLNGNLLTRLYPNE LKVMPFAGVLEHIGGIMEIQLEENP RPPKMRNRPTPRVTVSKDRQSFGPIMVY
Rat QLFLNGNLLTRLYPNE LKVMPFAGVLEHIGGIMEIQLEENP RPPKMRNRPTPRVTVSKDRQSFGPIMVY
Dog QLFLNGNLLTRLYPNE LKVMPFAGVLEHIGGIMEIQLEENP RPPKMRNRPTPRVTVSKDRQSFGPIMVY
Zebrafish QLFLNGNFLTKISPNE LKTLPFAGVLEHIGGIMEIQLEENP RPPKMRYRPTPRVTSSRDKHVFGPIMVY
Chicken QLFLNGNALTRLFPNE LKMMPFAGVLEHIGGIMEIQLEENP RPPKARNRPTPRVTVSKDRQIFGPIMVY
P374R
R426C
E461*
R484Q
Human NISDLQPKPTSPKKLY AFTNLTSLRRLYLNGN YLEYNVIKEIKPLTFD LFLNNNLLRSLPDNIF
Gorilla NISDLQPKPTSPKKLY AFTNLTSLRRLYLNGN YLEYNVIKEIKPLTFD LFLNNNLLRSLPDNIF
Macaque NISDLQPKPTSPKKLY AFTNLTSLRRLYLNGN YLEYNVIKEIKPLTFD LFLNNNLLRSLPDNIF
Bovine NISDLQPKPTSPKKLY AFTNLTSLRRLYLNGN YLEYNVIKEIKPLTFD LFLNNNLLRSLPDNIF
Sheep NISDLQPKPTSPKKLY AFTNLTSLRRLYLNGN YLEYNVIKEIKPLTFD LFLNNNLLRSLPDNVF
Mouse NISDLQPKPTSPKKLY AFTNLTSLRRLYLNGN YLEYNVIKEIKPLTFD LFLNNNLLRSLPDNIF
Rat NISDLQPKPTSPKKLY AFTNLTSLRRLYLNGN YLEYNVIKEIKPLTFD LFLNNNLLRSLPDNIF
Dog NISDLQPKPTSPKKLY AFTNLTSLRRLYLNGN YLEYNVIKEIKPLTFD LFLNNNLLRSLPDNIF
Zebrafish NISELQPKPSYPKKLH AFENLTSLRRLYLNGN YLEYNIIKEILPHTFN LFLNNNLLRSLPDNVF
Chicken NISELHPKPTTPKKLY AFTNLTSLRRLYLNGN YLEYNVIKEILPHTFD LFLNNNLLRSLPDNVF
V511M
E555D
V589I
R792C
Human NNHFSHLPVKGVLDQL ANSPVIINEVTCESPA LS---DGTVLSMNHNT APSYE-SRRQNQDRIN
Gorilla NNHFSHLPVKGVLDQL ANSPVIINEVTCESPA LS---DGTILSMNHNT APSYE-SRRQNQDRIN
Macaque NNHFSHLPVKGVLDQL ANSPVIINEVTCESPA LS---DGTILSMNHNT APSYE-SRRQNQDRIN
Bovine NNHFSHLPVKGVLDQL ANSPVIINEVTCESPA LS---DGTVLSMNHNT TPSYE-SRRQNQDRIN
Sheep NNHFSHLPVKGVLDQL ANSPVIINEVTCESPA LS---DGTVLSMNHNT TPSYE-SRRQNQDRIN
Mouse NNHFSHLPVKGVLDQL ANSPVIINEVTCESPA LS---DGTILSMNHNT APSYE-SRRQNQDRIN
Rat NNHFSHLPVKGVLDQL ANSPVIINEVTCESPA LS---DGTVLSMNHNT APSYE-SRRQNQDRIN
Dog NNHFSHLPVKGVLDQL ANSPVIINEVTCESPA LS---DGTVLSMNHNT APSYE-SRRQNQDRIN
Zebrafish NNHFSHLPVRGVLDQL SSTSVVVNEITCDSPS VSvtkPPTVVSSSTEA IPPYEtSRRLNQDRLN
Chicken NNHFSHLPVRGVLDRL SNPPVVINEVICESPT LS---ESSLLPMNQNT APSYE-SRRQNQDRIN

Supplementary Figure 2. Alignment and conservation across different species of SLITRK2 residues mutated in human patients.

a Alignment of human SLITRK1-6 amino acids surrounding SLITRK2 mutated residues found in human patients. The target mutated residues are indicated in yellow letters on a black background.

b Similarity or identity of mutated residues investigated in the current study across different species. The following GenBank accession numbers were utilized for sequence alignment: SLITRK1/human, NP_001268432.1; SLITRK2/human, NP_001137475.1; SLITRK3/human, NP_001305739.1; SLITRK4/human, NP_001171678.1; SLITRK5/human, NP_001371538.1; SLITRK6/human, NP_115605.2; Slitrk2/gorilla, XP_030861981.1; Slitrk2/macaque, XP_024649663.1; Slitrk2/bovine, XP_005227636.1; Slitrk2/sheep, XP_011962603.2; Slitrk2/mouse, XP_006528079.1; Slitrk2/rat, XP_006257688.1; Slitrk2/dog, XP_038306961.1; Slitrk2/zebrafish, XP_001332767.2; and Slitrk2/chicken, XP_040526579.1.

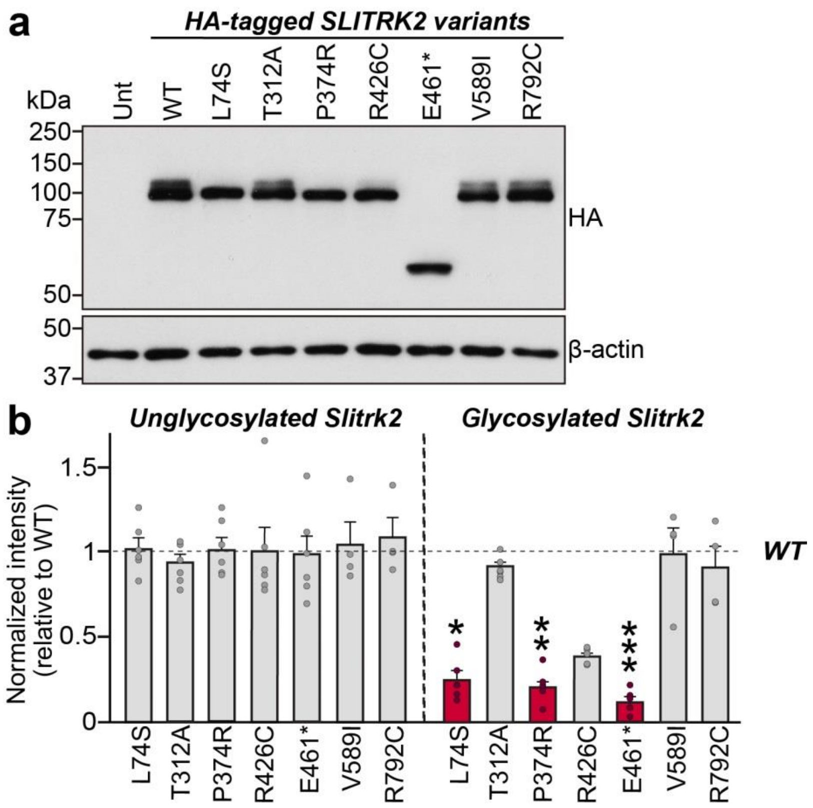

Supplementary Figure 3. Abnormally altered glycosylation of recombinant SLITRK2 mutant proteins.

a Representative immunoblots from HEK293T cells transfected with the indicated WT or mutant forms of SLITRK2. Samples containing equal amounts of protein were resolved by SDS-PAGE and immunoblotted using anti-HA; β-actin was used for normalization. Molecular mass markers are labeled in kilodaltons.

b Quantification of unglycosylated and glycosylated species of SLITRK2 WT protein or the indicated mutant forms. Data are mean ± SEMs ('n' denotes the number of independent experiments; WT, n = 10; L74S, n = 6; T312A, n = 6; P374R, n = 6; R426C, n = 6; E461*, n = 6; V589I, n = 4; and R792C, n = 4; *$p < 0.05$, **$p < 0.01$, ***$p < 0.001$; ANOVA with a non-parametric Kruskal-Wallis test). See **Source Data** for raw data values and **Supplementary Table 4** for statistical details.

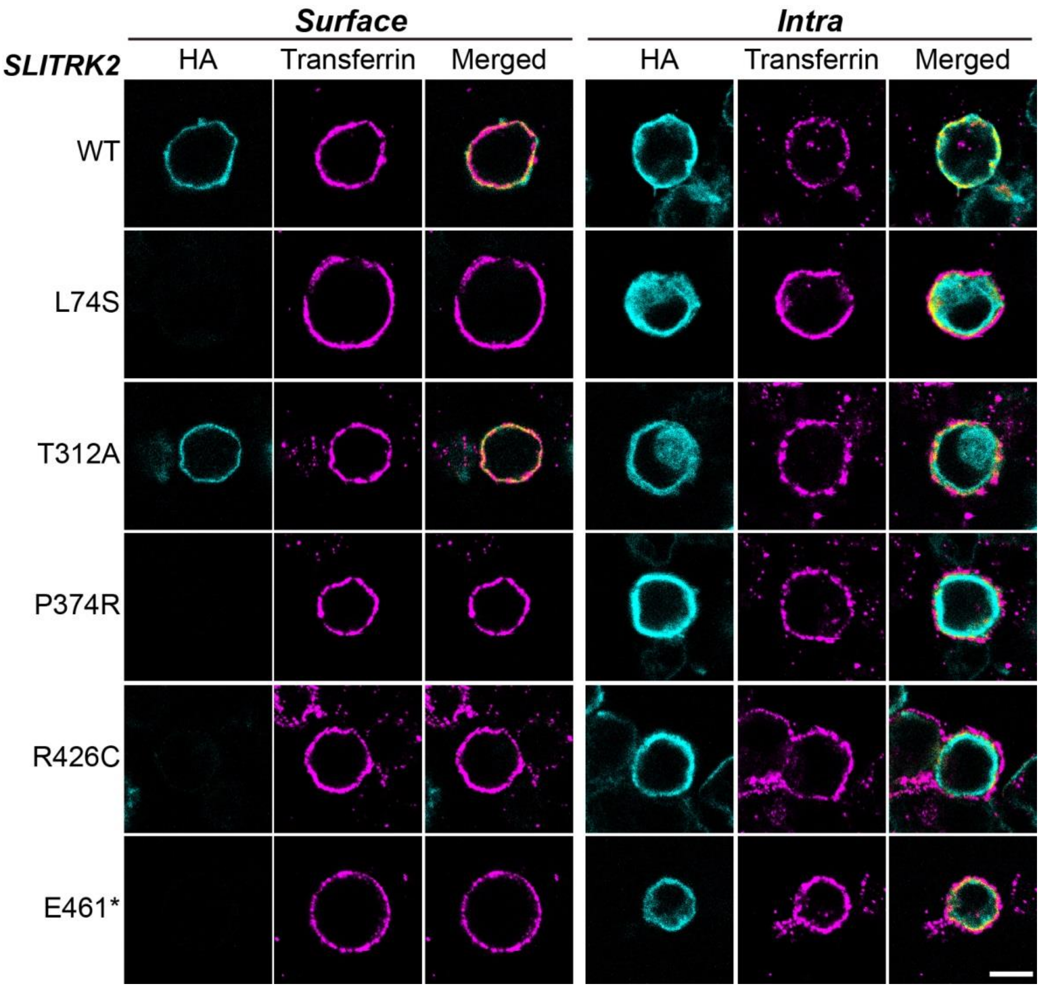

Supplementary Figure 4. Monitoring of surface expression of a subset of SLITRK2 variants in HEK293T cells by immunofluorescence analysis.

Surface expression analysis of HEK293T cells expressing the indicated WT or mutant forms of SLITRK2. Transfected HEK293T cells were immunostained with mouse anti-HA antibodies (cyan) and rabbit anti-transferrin antibodies (magenta) under non-permeabilized (surface) or permeabilized (intra) conditions. Scale bar, 10 μm (applies to all images). The experiments were independently repeated three times.

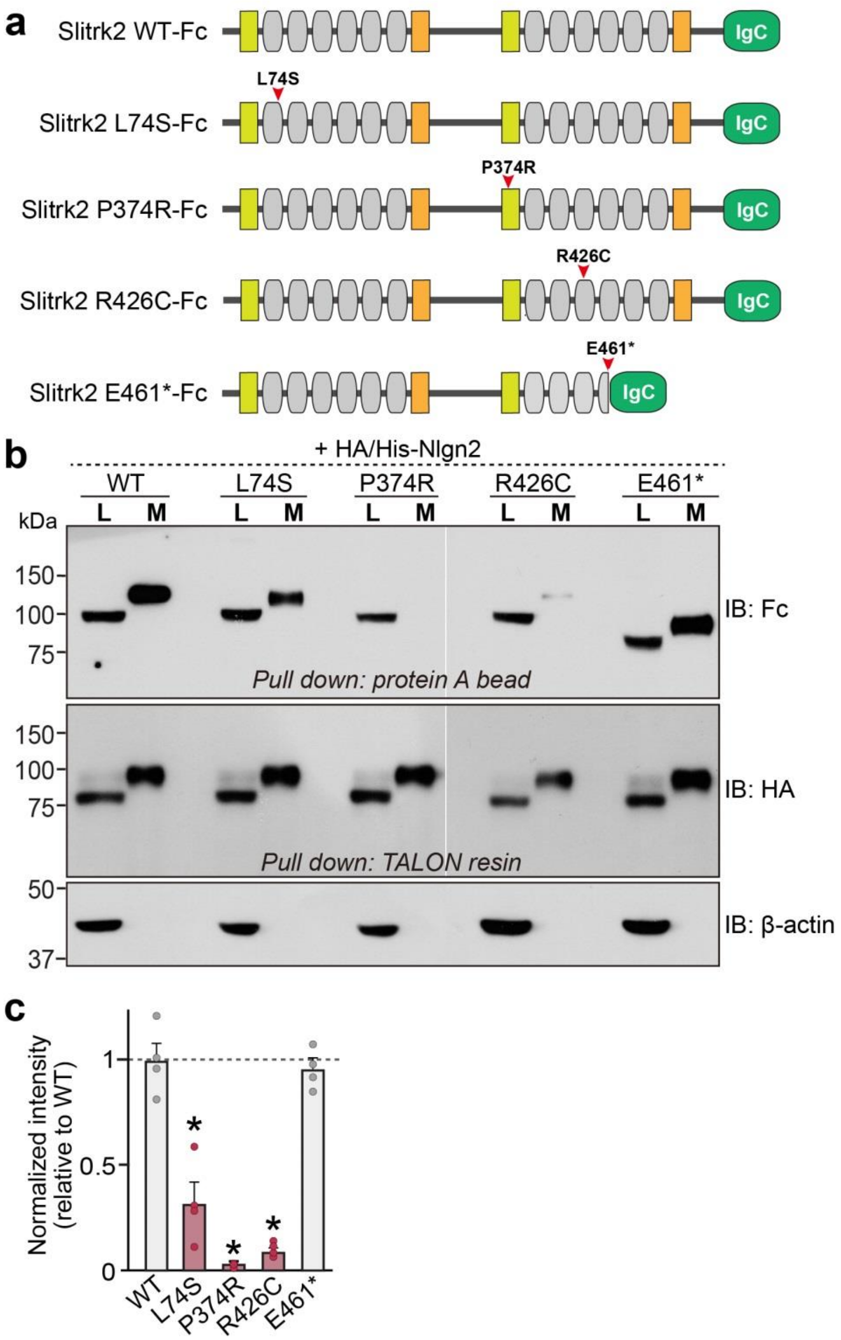

Supplementary Figure 5. Impaired secretion of recombinant SLITRK2 mutant proteins.

a Schematic diagrams depicting Ig-fused Slitrk2 WT and its variants.

b Representative images of western blots from lysates (L) or media (M) of HEK293T cells transfected with Ig-fused SLITRK2 and HA/His-tagged Nlgn2 vectors and immunoblotted with human Fc or HA antibodies.

c Quantification of secreted levels of the indicated SLITRK2-Fc recombinant proteins in HEK293T cells. Data are mean ± SEMs ('n' denotes the number of independent experiments; WT, n = 4; L74S, n = 4, P374R, n = 4; R426C, n = 4; and E461*, n = 4; *$p < 0.05$; two-tailed Mann-Whitney *U* test). See **Source Data** for raw data values and **Supplementary Table 4** for statistical details.

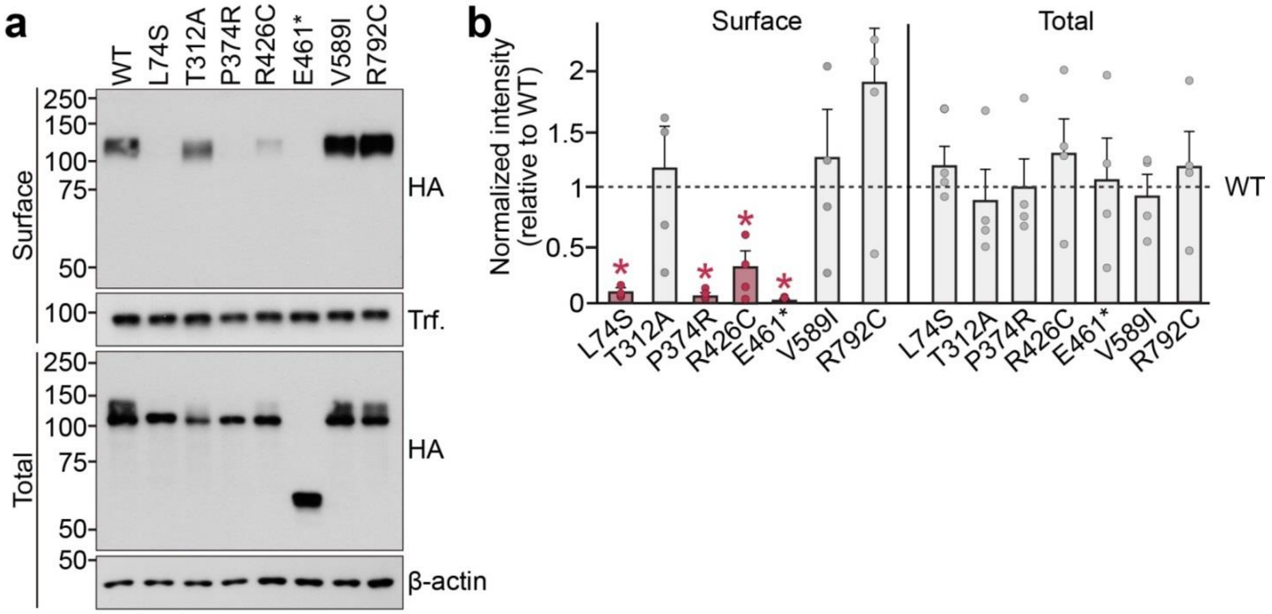

Supplementary Figure 6. Monitoring of surface expression of a subset of SLITRK2 variants in HEK293T cells by biotinylation analysis.

a Surface exposure of SLITRK2 variants on transfected HEK293T cells, analyzed by immunoblotting of affinity-purified, surface-biotinylated SLITRK2 proteins. Biotinylated cell surface proteins and total lysate proteins were assessed by immunoblot with anti-HA antibodies.

b Quantification of surface or total levels of SLITRK2 WT or the indicated mutant forms. Data are means ± SEMs ('n' denotes the number of independent experiments; n = 4 for all experimental groups; *****$p < 0.05$; two-tailed Mann-Whitney *U* test). See **Source Data** for raw data values and **Supplementary Table 4** for statistical details.

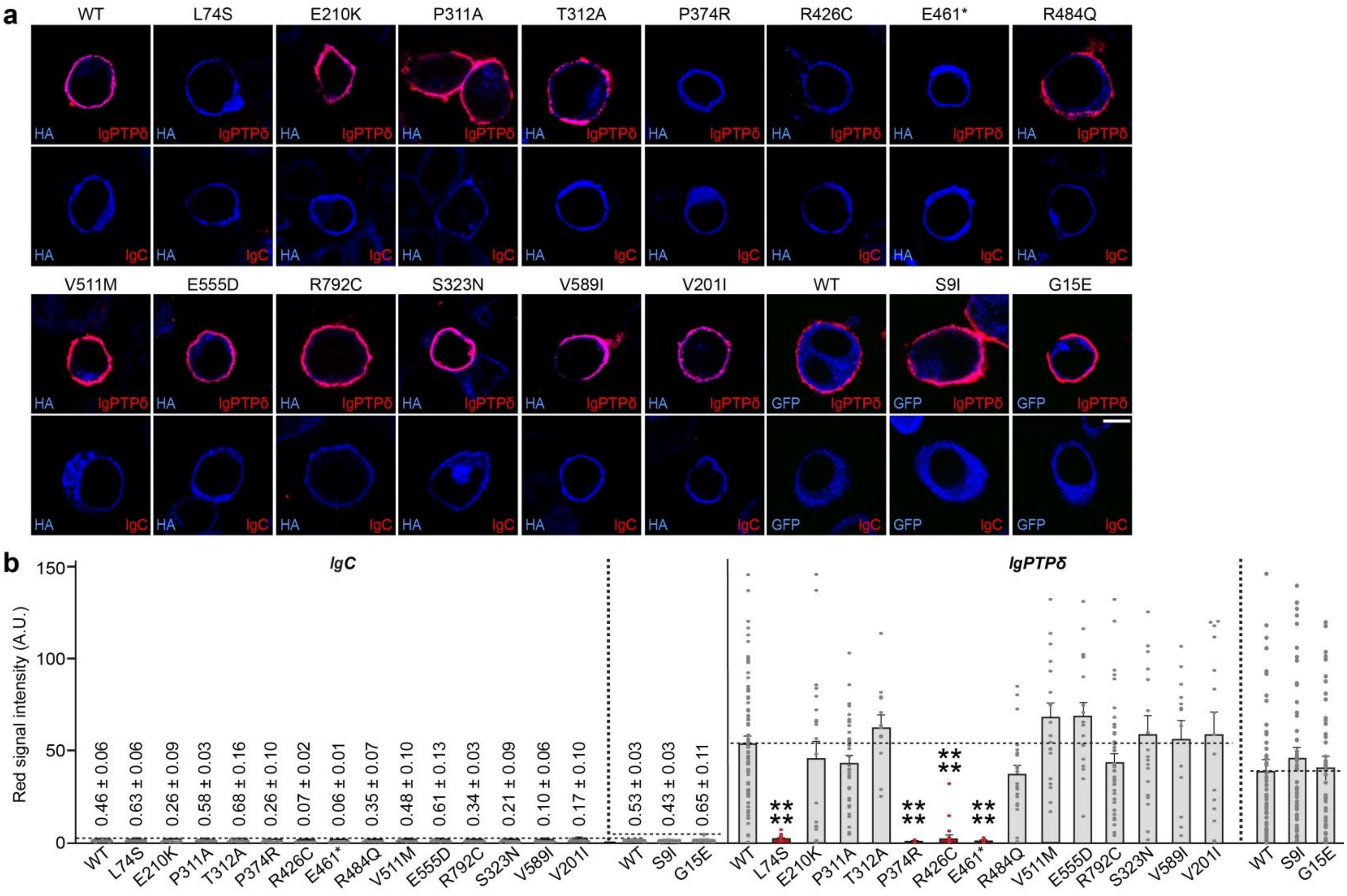

Supplementary Figure 7. Impaired PTPδ-binding of a subset of SLITRK2 variants.

a Representative images of cell-surface–binding assays. HEK293T cells expressing the indicated WT or variant forms of SLITRK2 were incubated with 10 μg/ml control IgC or Ig-PTPδ, and then analyzed by immunofluorescence imaging of Ig-fusion proteins (red) and HA antibodies (blue; pseudocolor). Scale bar, 10 μm (applies to all images).

b Quantification of cell surface binding in **a**. Data are mean ± SEMs ('n' denotes the number of cells from three independent experiments; IgC: WT, n = 34; L74S, n = 21; E210K, n = 10; P311A, n = 15; T312A, n = 8; P374R, n = 13; R426C, n = 7; E461*, n = 8; R484Q, n = 8 ; V511M, n = 9 ; E555D, n = 9; R792C, n = 14; S323N, n = 13; V589I, n = 11; V201I, n = 13; WT, n = 28; S9I, n = 29; and G15E, n = 30; IgPTPδ: WT, n = 74; L74S, n = 43; E210K, n = 22; P311A, n = 34 ; T312A, n = 12; P374R, n = 15; R426C, n = 20; E461*, n = 17; R484Q, n = 24; V511M, n = 21; E555D, n = 20; R792C, n = 42; S323N, n = 19; V589I, n =13; V201I, n = 15; WT, n = 51; S9I, n = 54; and G15E, n = 47; *****p* < 0.0001; ANOVA with non-parametric Kruskal-Wallis test). See **Source Data** for raw data values and **Supplementary Table 4** for statistical details.

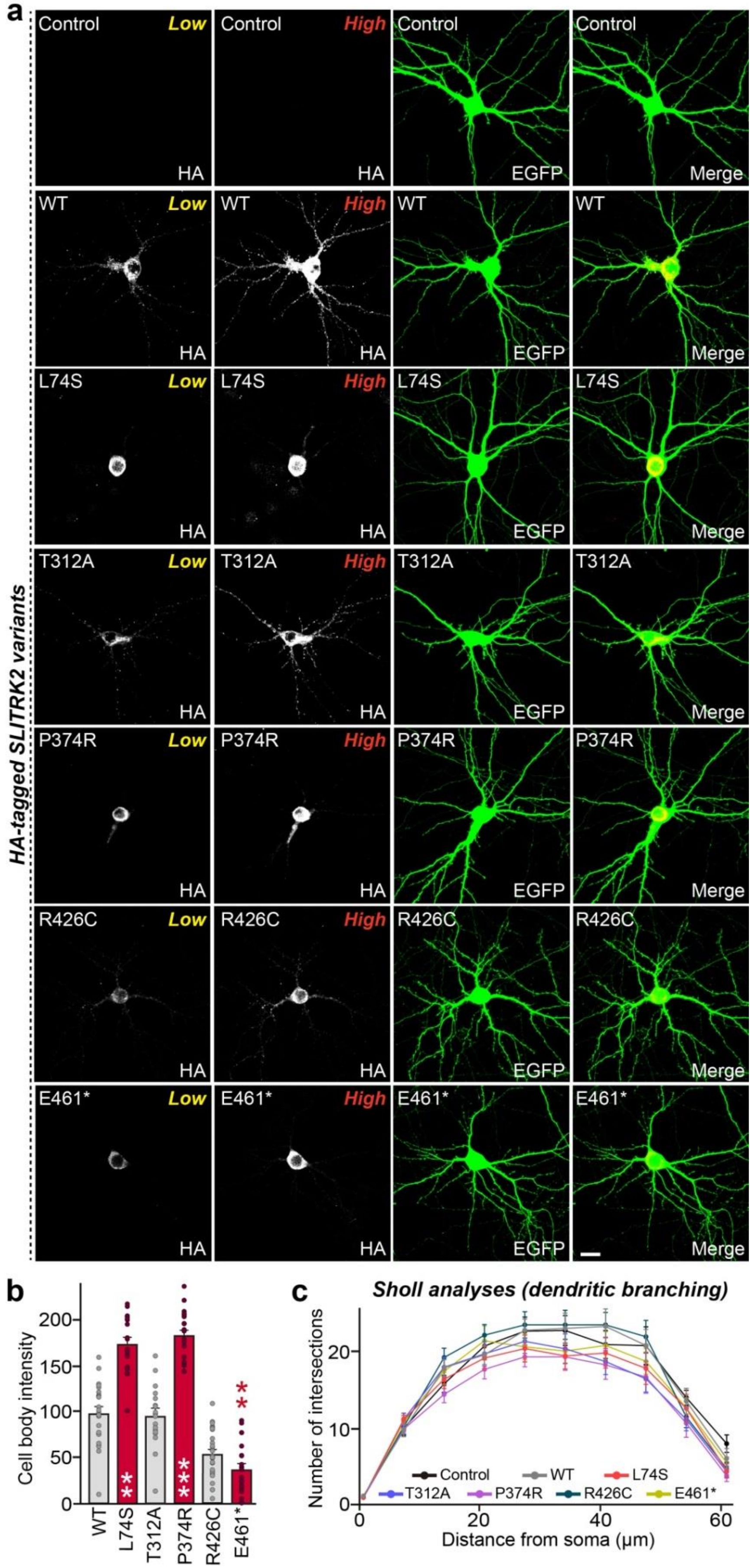

a
Control
Low
High
HA
EGFP
Merge
WT
L74S
T312A
P374R
R426C
E461*
HA-tagged SLITRK2 variants
b
Cell body intensity
WT
L74S
T312A
P374R
R426C
E461*
c
Sholl analyses (dendritic branching)
Number of intersections
Distance from soma (μm)
Control
WT
L74S
T312A
P374R
R426C
E461*

Supplementary Figure 8. Impaired dendritic targeting of a subset of SLITRK2 variants in cultured hippocampal neurons.

a Representative images of hippocampal neurons transfected at DIV10 with the indicated WT or mutant forms of SLITRK2 together with EGFP vector. Transfected neurons were double-immunostained at DIV14 with antibodies against HA (gray) and EGFP (green). Images were captured by confocal microscopy using different exposure times (low or high). Scale bar, 20 μm (applies to all images).

b Quantification of the average intensity of HA immunoreactivity in the soma of transfected neurons using images acquired at low exposure times. Data are shown as means ± SEMs ('n' denotes the number of neurons from three independent experiments; WT, n = 24; L74S, n = 19; T312A, n = 18; P374R, n = 22; R426C, n = 28; and E461*, n = 22; ***p* < 0.01, ****p*< 0.001; ANOVA with non-parametric Kruskal-Wallis test).

c Quantification of dendritic branching in cultured neurons by Sholl analysis demonstrating no alterations in neurons expressing the indicated SLITRK2 variants. Data are shown as means ± SEM ('n' denotes the number of neurons from three independent experiments; Control, n = 14; WT, n = 24; L74S, n = 25; T312A, n = 18; P374R, n = 25; R426C, n = 21; and E461*, n = 22). See **Source Data** for raw data values and **Supplementary Table 4** for statistical details.

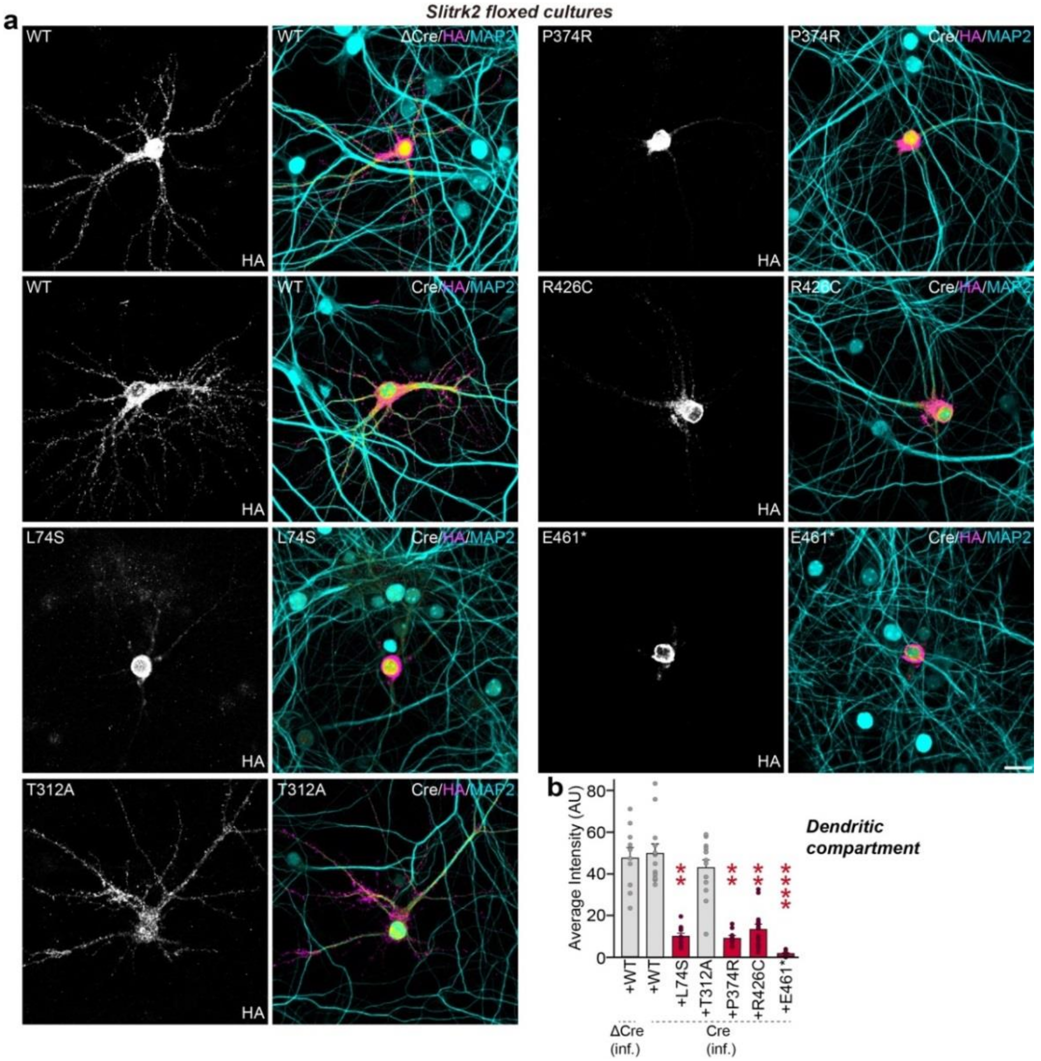

Supplementary Figure 9. Impaired dendritic targeting of a subset of SLITRK2 variants in *Slitrk2*-cKO hippocampal neurons.

a Representative images of cultured hippocampal neurons (DIV14) from Slitrk2$^{f/f}$ mice infected with lentiviruses expressing ΔCre or Cre at DIV5 and transfected with vectors expressing SLITRK2 WT or the indicated mutant forms at DIV8–9. Neurons were immunostained using antibodies against HA (magenta), and MAP2 (cyan [non-nuclear fluorescence signal]). Scale bar, 20 μm (applies to all images).

b Dendritic targeting of SLITRK2 WT or the indicated mutant forms in *Slitrk2*-cKO hippocampal cultured neurons was quantified by measuring average intensity of HA immunofluorescence in primary dendrites. Data are shown as means ± SEMs ('n' denotes the number of neurons from three independent batches; ΔCre+WT, n = 10; Cre+WT, n = 13; Cre+L74S, n = 12, Cre+T312A, n = 13, Cre+P374R, n = 10; Cre+R426C, n = 16; and Cre+E461*, n = 11; $**p < 0.01$, $****p < 0.0001$; ANOVA with non-parametric Kruskal-Wallis test). See **Source Data** for raw data values and **Supplementary Table 4** for statistical details.

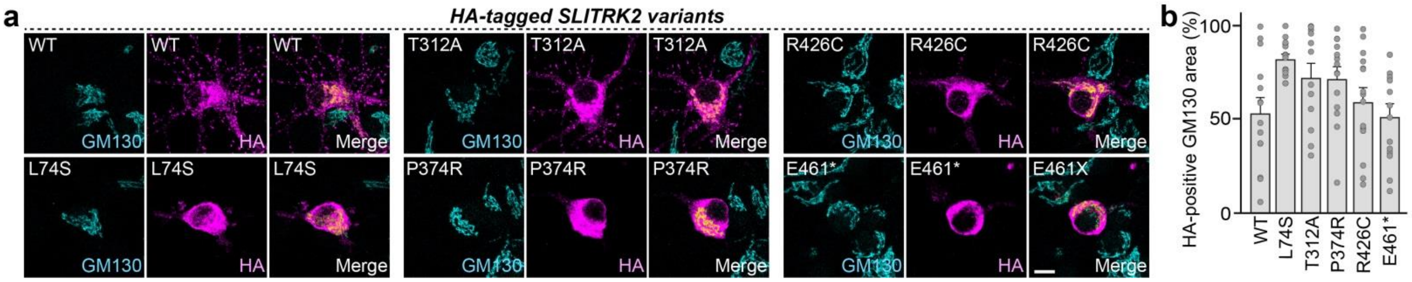

Supplementary Figure 10. Analysis of the distribution of a subset of recombinant SLITRK2 variants in the *cis*-Golgi compartment of cultured neurons.

a Representative images of hippocampal cultured neurons transfected with SLITRK2 WT or the indicated mutant forms at DIV10. Transfected neurons were double-immunostained with antibodies against the *cis*-Golgi marker GM130 (cyan) and HA (magenta) at DIV14. Scale bar, 20 μm (applies to all images).

b Quantification of HA immunoreactive-positive GM130 puncta area. Data are means ± SEMs (‘n’ denotes the number of neurons from three independent experiments; WT, n = 14; L74S, n = 16, T312A, n = 12; P374R, n = 14; R426C, n = 15; and E461*****, n = 14). See **Source Data** for raw data values and **Supplementary Table 4** for statistical details.

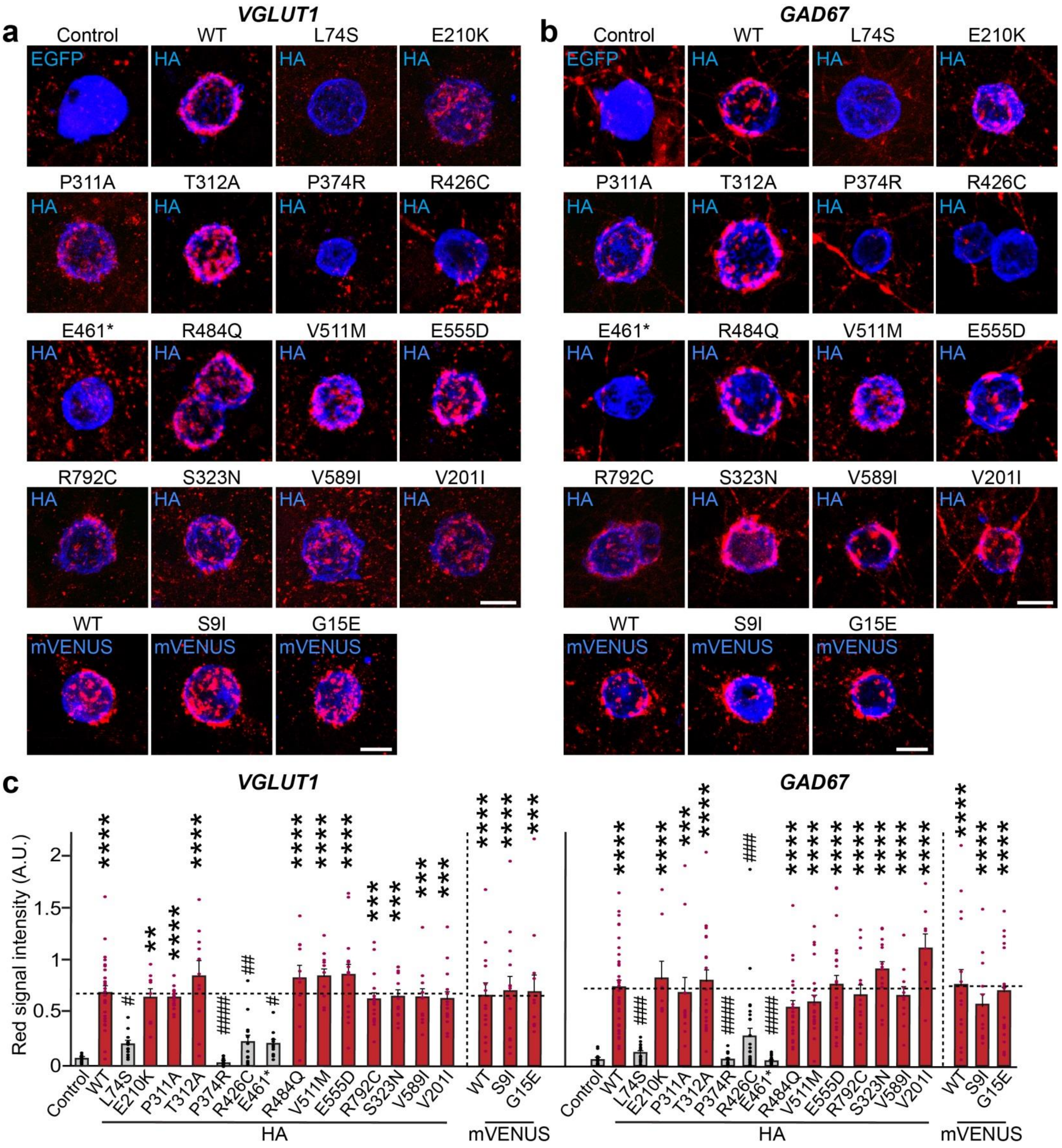

VGLUT1
GAD67
a
b
Control
WT
L74S
E210K
EGFP
HA
P311A
T312A
P374R
R426C
E461*
R484Q
V511M
E555D
R792C
S323N
V589I
V201I
S9I
G15E
mVENUS
c
Red signal intensity (A.U.)
Control
HA

Supplementary Figure 11. Impaired synaptogenic activity of a subset of SLITRK2 variants in cultured neurons.

a, b Representative images of the heterologous synapse-formation activities of WT SLITRK2 and the indicated point mutants in cultured hippocampal neurons. Neurons were cocultured with HEK293T cells transfected with the indicated WT or variant forms of SLITRK2 at DIV10. Neurons were then immunostained at DIV12 with antibodies against EGFP or HA (blue) and VGLUT1 (**a**; red) or GAD67 (**b**; red). Scale bar, 10 μm (applies to all images).

c Synapse-formation activity was quantified by measuring the ratio of VGLUT1 or GAD67 staining intensity (red) to HA/EGFP intensity (blue). All data are shown as means ± SEMs ('n' denotes the number of cells from three independent batches; VGLUT1: Control, n = 17; WT, n = 28; L74S, n = 13; E210K, n = 10; P311A, n = 15; T312A, n = 11; P374R, n = 15; R426C, n = 15; E461*, n = 12; R484Q, n = 11; V511M, n = 11; E555D, n = 15; R792C, n = 16; S323N, n = 13; V589I, n = 12; V201I, n = 15; WT, n = 14; S9I, n = 14; and G15E, n = 12; GAD67: Control, n = 29; WT, n = 36; L74S, n = 16; E210K, n = 8; P311A, n = 12; T312A, n = 21; P374R, n = 15; R426C, n = 27; E461*, n = 21; R484Q, n = 22; V511M, n = 21; E555D, n = 23; R792C, n = 13; S323N, n = 14; V589I, n =11; V201I, n = 9; WT, n = 16; S9I, n = 13; and G15E, n = 18; $^{**}p < 0.01$, $^{***}p < 0.001$, $^{****}p < 0.0001$; Control vs. experimental group; $^{\#}p < 0.05$, $^{\#\#}p < 0.01$, $^{\#\#\#}p < 0.001$, $^{\#\#\#\#}p < 0.0001$ WT vs. experimental group; ANOVA with a non-parametric Kruskal-Wallis test). See **Source Data** for raw data values and **Supplementary Table 4** for statistical details.

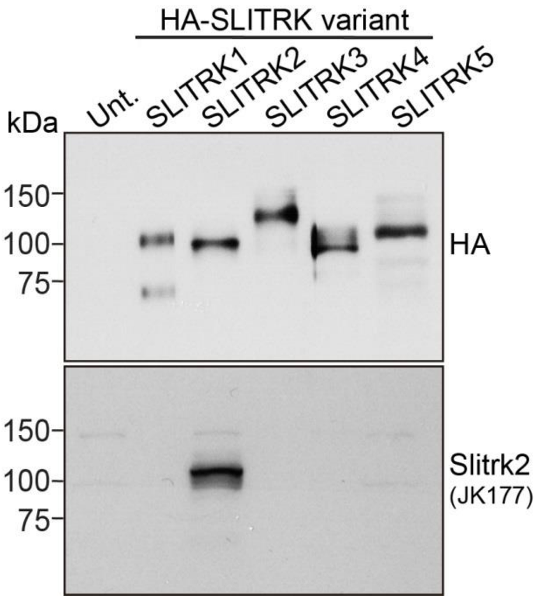

Supplementary Figure 12. Cross-reactivity of the in-house SLITRK2 antibody.

Lysates of untransfected (Unt.) or transfected HEK293T cells expressing the indicated SLITRK expression plasmids were immunoblotted with the anti-Slitrk2 antibody JK177. Expression of HA-tagged SLITRK plasmids was verified by immunoblotting with anti-HA antibodies. The experiments were independently repeated three times.

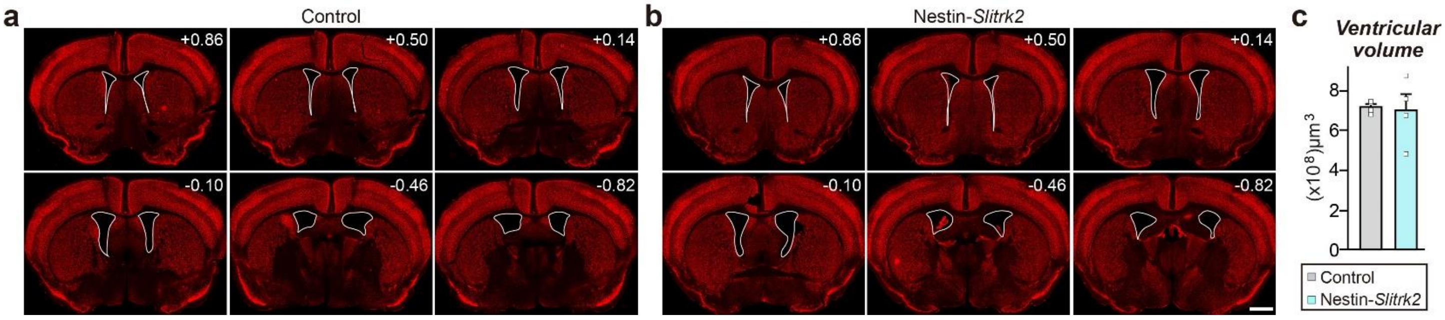

Supplementary Figure 13. Ventricular volumes are comparable between control and *Slitrk2*-cKO mice.

a, b Representative images of NeuN-stained coronal sections of Control (**a**) and Nestin-*Slitrk2* (**b**) mice. Scale bar, 1 mm (applies to all images).

c Summary data showing quantification of ventricular volume. Data are means ± SEMs (Ctrl, n = 4 mice; Nestin-*Slitrk2*, n = 4 mice). See **Source Data** for raw data values and **Supplementary Table 4** for statistical details.

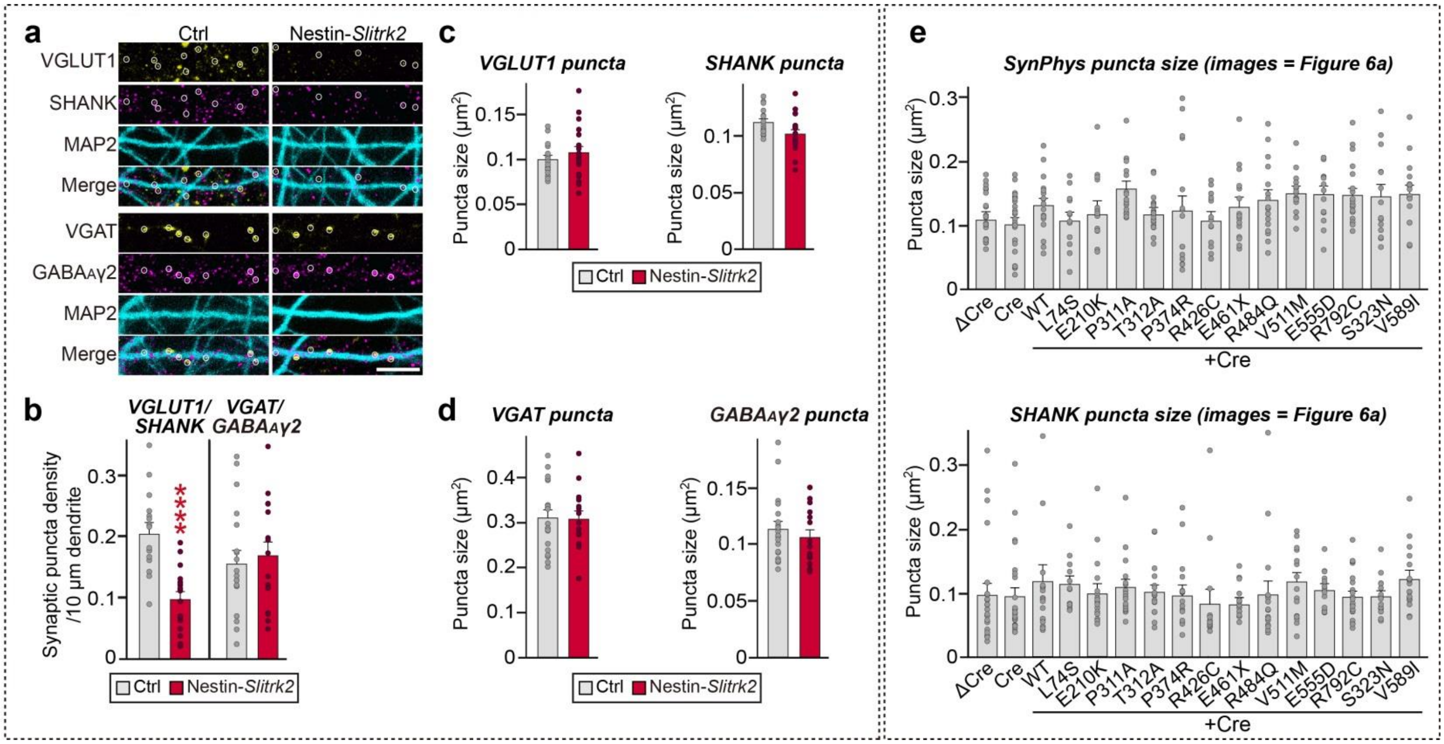

Supplementary Figure 14. Impaired excitatory synapse maintenance in cultured hippocampal neurons from Nestin-*Slitrk2* mice.

a Representative images from Nestin-*Slitrk2* cultured hippocampal neurons using antibodies against MAP2 (cyan), VGLUT1 (yellow), SHANK (magenta), VGAT (yellow) and $GABA_A\gamma2$ (magenta) at DIV14. Scale bar, 10 μm (applies to all images).

b Summary data showing quantification of excitatory and inhibitory synaptic puncta density. Data are means ± SEMs ('n' denotes the number of neurons from three independent batches; VGLUT1/SHANK/Ctrl, n = 16; VGLUT1/SHANK/Nestin-*Slitrk2*; n = 19; VGAT/$GABA_A\gamma2$/Ctrl, n = 19; and VGAT/$GABA_A\gamma2$/Nestin-*Slitrk2*, n = 15; ****$p < 0.0001$, two-tailed Mann-Whitney *U* test).

c, d Summary data showing quantification of excitatory (**c**) and inhibitory (**d**) synaptic puncta size. Data are means ± SEMs ('n' denotes the number of neurons from three independent batches; VGLUT1/SHANK/Ctrl, n = 16; VGLUT1/SHANK/Nestin-*Slitrk2*; n = 19; VGAT/$GABA_A\gamma2$/Ctrl, n = 19; and VGAT/$GABA_A\gamma2$/Nestin-*Slitrk2*, n = 15; two-tailed Mann-Whitney *U* test).

e Summary data showing quantification of excitatory synaptic puncta size in cultured Slitrk2-floxed hippocampal neurons transfected with the indicated SLITRK2 variants. Data are means ± SEMs. See **Figure 6** for representative images and quantification of excitatory synaptic puncta density. See **Source Data** for raw data values and **Supplementary Table 4** for statistical details.

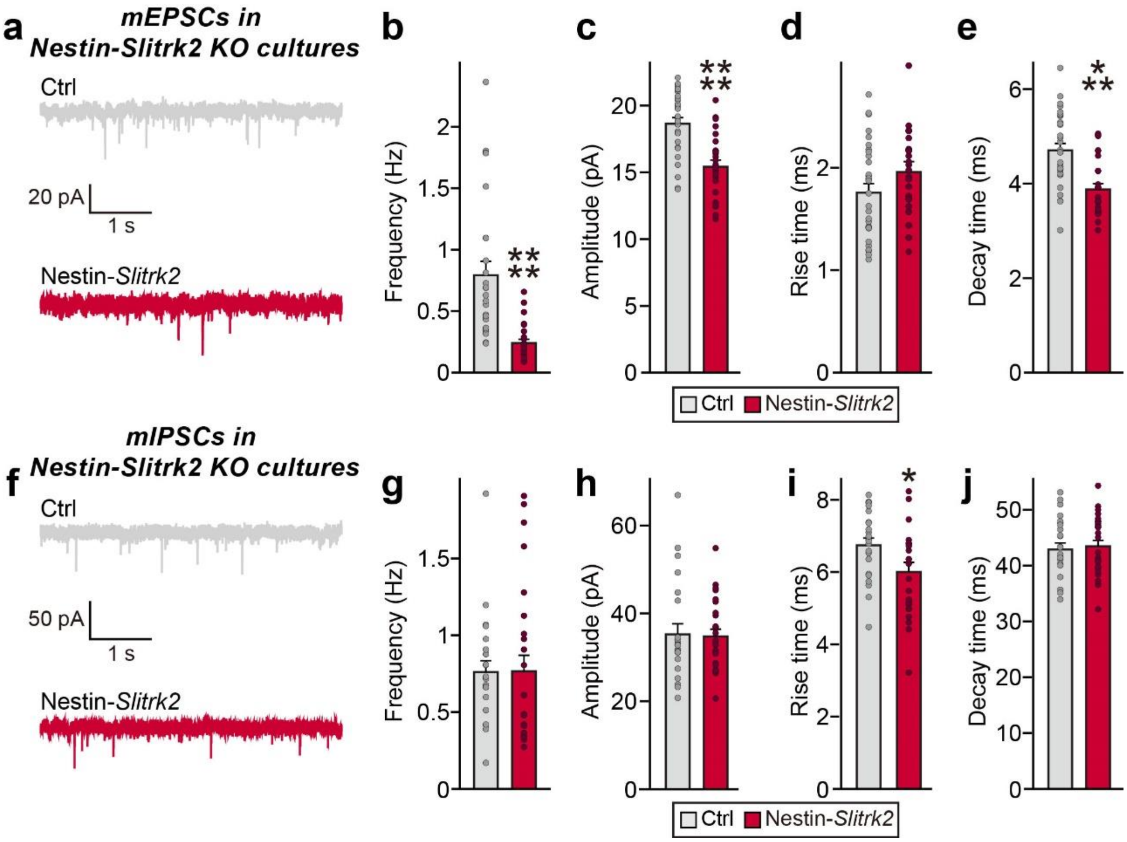

Supplementary Figure 15. Impaired excitatory synaptic transmission in cultured hippocampal neurons from Nestin-*Slitrk*2 mice. a–e Representative mEPSC traces (**a**) and quantification of frequency (**b**), amplitudes (**c**), rise time (**d**), and decay time (**e**) of mEPSCs recorded in cultured hippocampal neurons from Nestin-*Slitrk*2 mice. Data are shown as means ± SEMs ('n' denotes the number of neurons from three independent experiments; Control, n = 26; Nestin-*Slitrk*2, n = 24; ***$p < 0.001$, ****$p < 0.0001$; two-tailed Mann-Whitney *U* test). **f–j** Representative mIPSC traces (**f**) and quantification of frequency (**g**), amplitude (**h**), rise time (**i**), and decay time (**j**) of mIPSCs recorded in cultured hippocampal neurons from Nestin-*Slitrk*2 mice. Data are shown as means ± SEMs ('n' denotes the number of neurons from three independent experiments; Control, n = 23; Nestin-*Slitrk*2, n = 25; *$p < 0.05$; two-tailed Mann-Whitney *U* test). See **Source Data** for raw data values and **Supplementary Table 4** for statistical details.

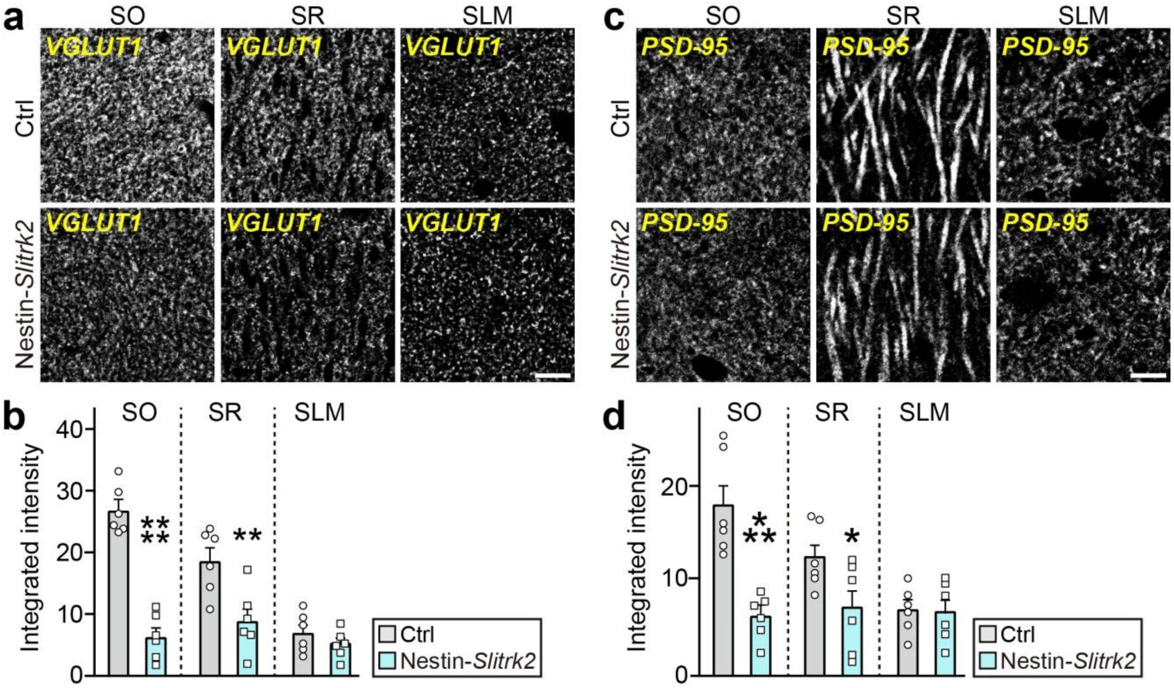

Supplementary Figure 16. Impaired excitatory synapse development in the hippocampal CA1 region of Nestin-*Slitrk2* mice.

a, c Representative images of hippocampal CA1 layers (SO, SR and SLM) in control and Nestin-*Slitrk2* mice, analyzed by assessing the excitatory synaptic markers VGLUT1 (**a**) or PSD-95 (**c**). Scale bar, 20 µm (applies to all images).

b, d Quantification of the integrated intensity of VGLUT1-positive (**b**) and PSD-95-positive (**d**) synaptic puncta. Data are shown as means ± SEMs (n = 6 mice; **p* < 0.05, ***p* < 0.01, ****p* < 0.001, *****p* < 0.0001; two-tailed unpaired t test). See **Source Data** for raw data values and **Supplementary Table 4** for statistical details.

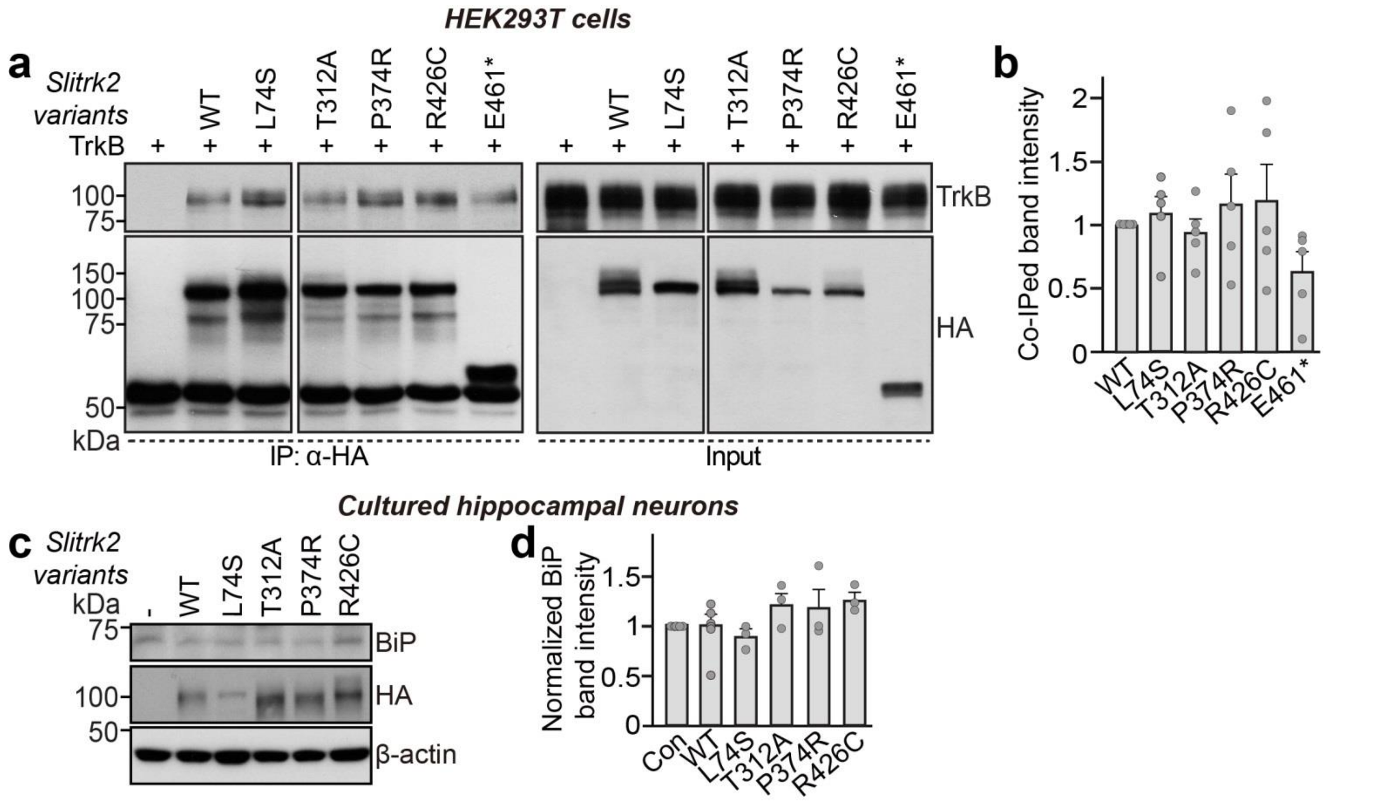

Supplementary Figure 17. No changes in TrkB binding and unfolded protein responses in cultured neurons expressing disease-associated SLITRK2 variants.

a Representative immunoblots showing co-immunoprecipitation of TrkB with SLITRK2 WT or the indicated SLITRK2 variants. HEK293T cells were transfected with HA-tagged SLITRK2 WT or its variants alone or together with untagged TrkB, after which coimmunoprecipitation of TrkB2 with SLITRK2 was assayed. Input, 5%.

b Quantification of coimmunoprecipitated TrkB in **a**, normalized to WT. Data are means ± SEMs (n = 5 independent experiments).

c Cultured cortical neurons were infected with lentiviruses expressing SLITRK2 WT or the indicated SLITRK2 variants. Levels of BiP were analyzed by semi-quantitative immunoblotting.

d Quantification of BiP levels from **c**. Data are means ± SEMs (WT, n = 6; L74S, n = 6; T312A, n = 3; P374R, n = 3; R426C, n = 3; and E461*, n = independent experiments). See **Source Data** for raw data values and **Supplementary Table 4** for statistical details.

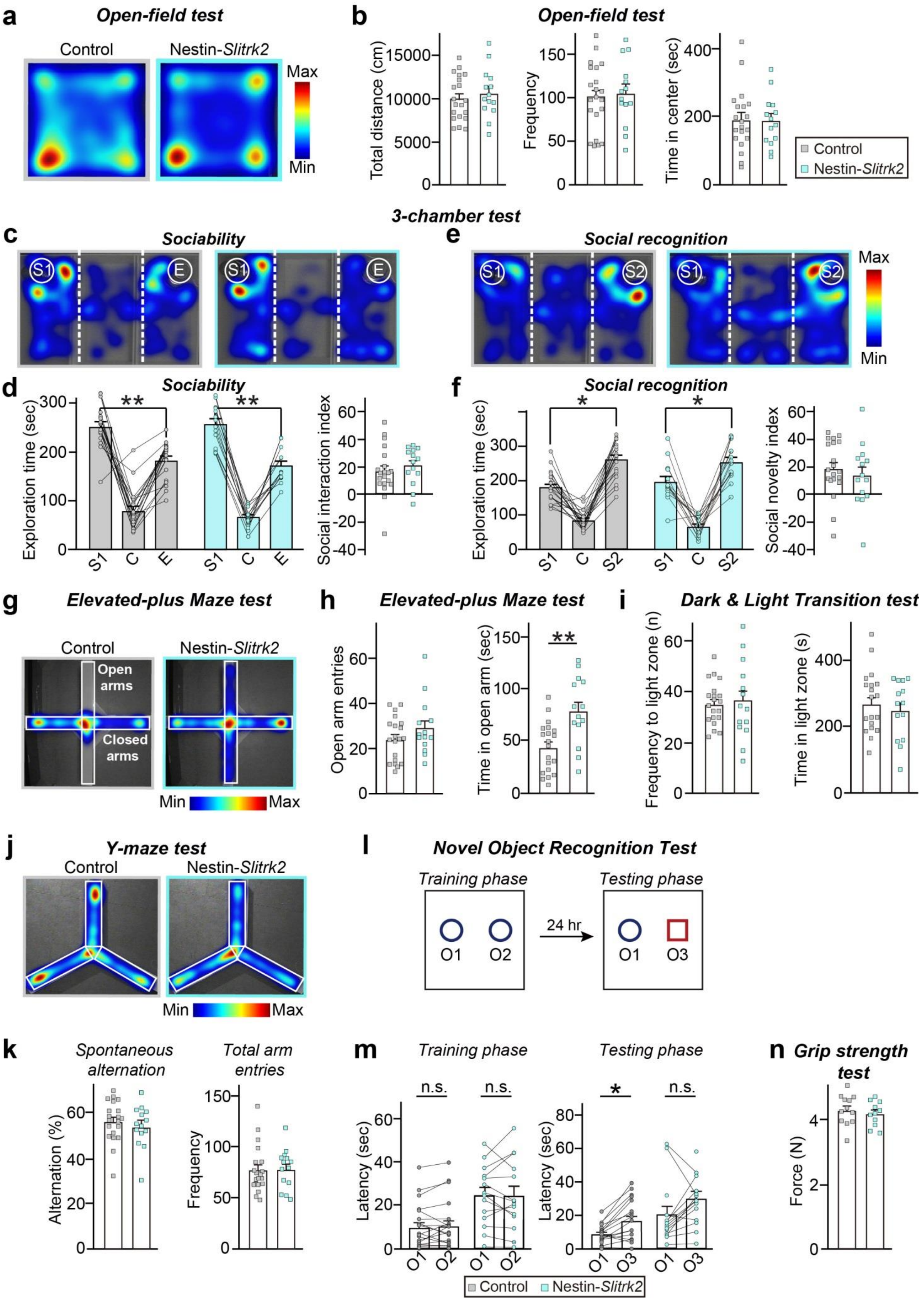

a
Open-field test
Control
Nestin-Slitrk2
Max
Min
b
Open-field test
Total distance (cm)
Frequency
Time in center (sec)
Control
Nestin-Slitrk2
3-chamber test
c
Sociability
S1
E
e
Social recognition
S2
d
Sociability
Exploration time (sec)
Social interaction index
f
Social recognition
Social novelty index
g
Elevated-plus Maze test
Open arms
Closed arms
h
Elevated-plus Maze test
Open arm entries
Time in open arm (sec)
i
Dark & Light Transition test
Frequency to light zone (n)
Time in light zone (s)
j
Y-maze test
l
Novel Object Recognition Test
Training phase
Testing phase
24 hr
O1
O2
O3
k
Spontaneous alternation
Total arm entries
Alternation (%)
m
Latency (sec)
n.s.
n
Grip strength test
Force (N)

Supplementary Figure 18. *Slitrk2*-cKO mice display anxiolytic behavior, with normal locomotion and social recognition.

a, b Analysis of locomotor activity by open field test in Control and Nestin-*Slitrk2* mice. (**a**) Representative heat maps of the time spent in the open chamber. (**b**) Number of entries into the center zone, time spent in the center zone, and total distance moved. Data are presented as means ± SEMs ('n' denotes the number of mice; Control, n = 20; Nestin-*Slitrk2*, n = 14).

c, d Analysis of sociability in Control and Nestin-*Slitrk2* mice by 3-chamber test. (**c**) Representative heat maps of the time spent in each side chamber containing a novel mouse (S1) or an empty wire cup (E), or time spent in the center chamber. (**d**) Summary graphs showing time in chamber and preference index. The preference index obtained from exploration time represents the numerical difference between the time spent exploring or sniffing the two targets (S1/stranger vs. E/empty wire cup) divided by total exploration time × 100. Data are presented as means ± SEMs ('n' denotes the number of mice; Control, n = 20; Nestin-*Slitrk2*, n = 14; $^{***}p < 0.001$; Tukey's multiple comparisons and Mann Whitney *U* test).

e, f Analysis of social novelty recognition in Control and Nestin-*Slitrk2* mice by 3-chamber test. (**e**) Representative heat maps of the time spent in each side chamber containing the previous mouse (S1) or a novel mouse (S2), or time spent in the center chamber. (**f**) Summary graphs showing time in chamber and preference index. The preference index obtained from exploration time represents the numerical difference between the time spent exploring or sniffing the two targets (S2/new stranger vs. S1/previous stranger) divided by total exploration time × 100. Data are presented as means ± SEMs ('n' denotes the number of mice; Control, n = 20; Nestin-*Slitrk2*, n = 14; $^{*}p < 0.05$; Tukey's multiple comparisons and Mann Whitney *U* test).

g, h Analysis of anxiety/exploration-related behavior by elevated plus maze (EPM) test in Control and Nestin-*Slitrk2* mice. (**g**) Representative heat maps of the time spent in the open arms of the EPM. Red represents increased time spent, and blue represents minimal time spent during the test. (**h**) Number of open arm entries and time spent in open arms. Data are presented as means ± SEMs ('n' denotes the number of mice; Control, n = 20; Nestin-*Slitrk2*, n = 14;$^{**}p < 0.01$; two-tailed Mann Whitney *U* test).

i Analysis of anxiety/exploration-related behavior by light-dark test (LDT) in Control and Nestin-*Slitrk2* mice. Number of entries into the light chamber and time spent in the light chamber are shown. Data are presented as means ± SEMs ('n' denotes the number of mice; Control, n = 20; Nestin-*Slitrk2*, n = 14).

j, k Analysis of spatial working memory in Control and Nestin-*Slitrk2* mice using a Y-maze test. Representative heat map showing movements of the indicated mice during the Y-maze test (**j**). Red represents increased time spent, and blue represents minimal time spent during the test. (**k**) Total number of arm entries and spontaneous alternation performance ratio (% of spontaneous alternations). Data are presented as means ± SEMs ('n' denotes the number of mice; Control, n = 20; Nestin-*Slitrk2*, n = 14).

l Schematic depiction of novel object-recognition memory test.

m Analysis of novel object recognition (NOR) memory in Control and Nestin-*Slitrk2* mice. Exploration time and novel object preference for 24-h testing phases. Mice were allowed to explore two identical objects (denoted O1 and O2), and after a 24-h delay were exposed to two different objects—one familiar object from the training phase (O1) and one novel object (O3). Data are presented as means ± SEMs ('n' denotes the number of mice; Control, n = 12; Nestin-*Slitrk2*, n = 11; $^{*}p < 0.05$; two-tailed paired t test).

n Test of grip strength in Nestin-*Slitrk2* and control mice. Data are presented as means ± SEMs ('n' denotes the number of mice; Control, n = 12; Nestin-*Slitrk2*, n = 11). See **Source Data** for raw data values and **Supplementary Table 4** for statistical details.

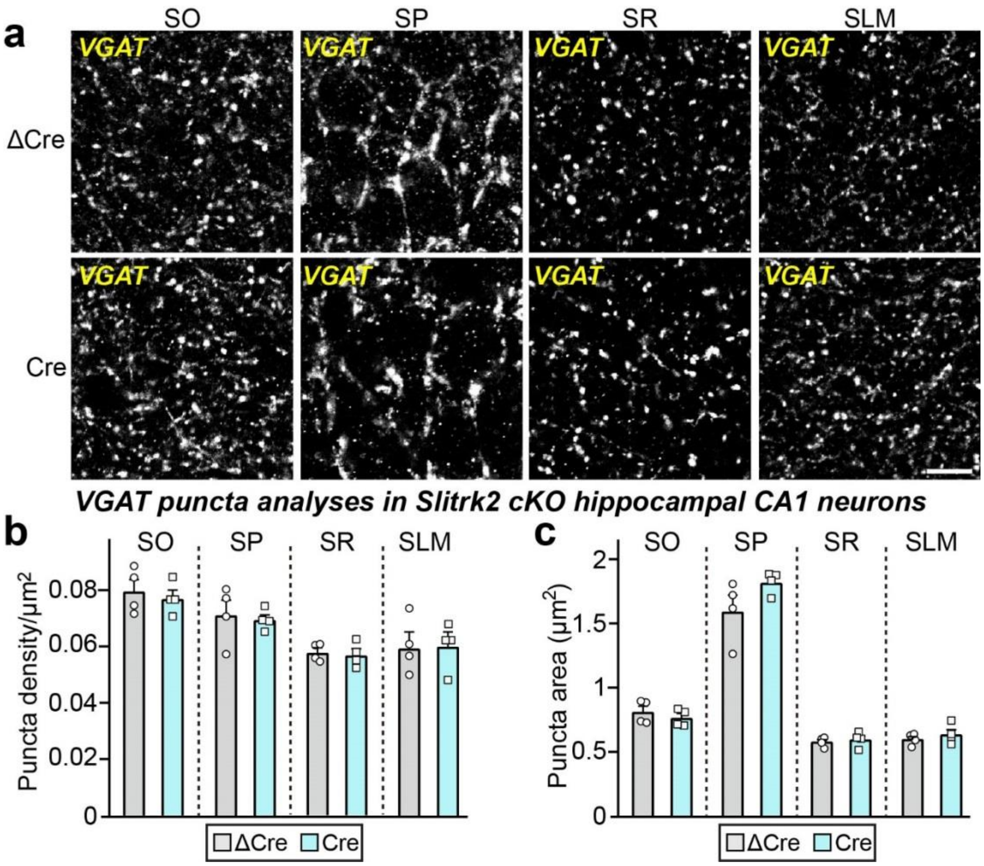

Supplementary Figure 19. No alterations in inhibitory synapse maintenance in hippocampal CA1-specific Slitrk2-cKO mice.

a Representative images showing VGAT puncta in hippocampal CA1 layers (SO, SP, SR and SLM) of *Slitrk2*$^{f/f}$ mice injected with AAVs expressing Cre or ΔCre. Scale bar, 20 μm (applies to all images).

b, c Quantification of VGAT puncta density (**b**) or size (**c**). Data are shown as means ± SEMs (n = 4 mice each after averaging data from 3 sections/mouse). See **Source Data** for raw data values and **Supplementary Table 4** for statistical details.

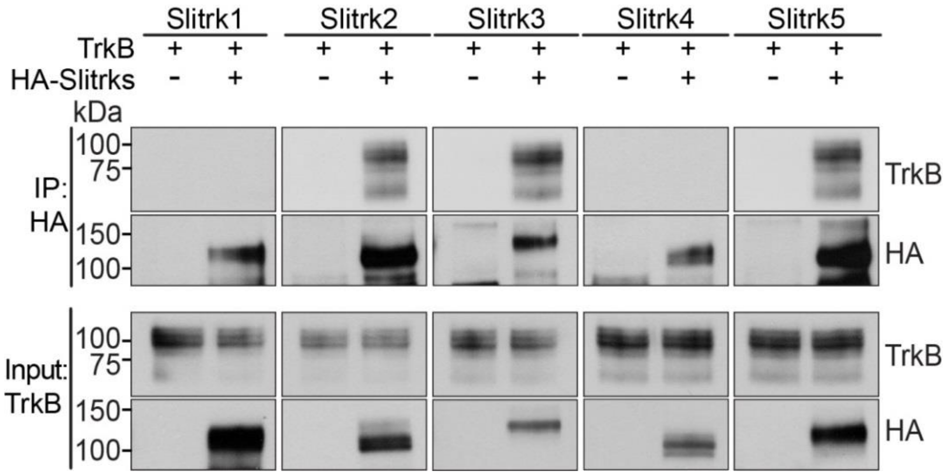

Supplementary Figure 20. TrkB interacts with Slitrk2, Slitrk3 and Slitrk5 in heterologous cells, but not with Slitrk1 or Slitrk4.

HEK293T cells were singly transfected with the indicated HA-tagged Slitrk expression plasmids or co-transfected with a TrkB expression plasmid, after which coimmunoprecipitation of Slitrks with TrkB was assayed. Input, 5%. The experiments were independently repeated three times.

Supplementary Tables

Table S1. ***SLITRK2*** **variants identified in the current study**

Variants are based on transcript NM_032539.4. PPH2, polyphen2; CADD, Combined Annotation Dependent Depletion; M, male; F, female; ML/DT, membrane localization (**Fig. 3**) and dendritic targeting (**Fig. 4**); I-PTPδ, interaction with PTPδ (**Fig. S4**); SF: synapse formation (**Fig. S5**); Res-Syn, impaired rescue of synaptic maintenance and activity (**Fig. 6**); NA, non-available; ACMG/AMP criteria, criteria for variant interpretation according to the American College of Medical Genetics and Genomics/Association for Molecular Pathology; Criteria supporting pathogenicity: PM2, absent from controls (in gnomAD); PS2, *de novo* in a patient with the disease and no family history; PS3, well-established *in vitro* or *in vivo* functional studies supportive of a damaging effect on the gene or gene product; PP1, cosegregation with disease in multiple affected family members in a gene definitively known to cause the disease; PP3, multiple lines of computational evidence support a deleterious effect on the gene or gene product; Criteria supporting a benign effect: BS2, observed in a healthy adult individual with full penetrance expected at an early age; BP4, multiple lines of computational evidence suggest no impact on the gene or gene product. a, absent from an unaffected maternal uncle; b, amino acid change not reported in gnomAD, whereas p.R426H was observed in a hemizygous state in one male; c, this individual carried a pathogenic variant in *CUL4B* which seemed to be sufficient to explain the majority of his clinical phenotypes.

Abbreviations: dam, damaging; Delet., deleterious; Extracell., extracellular; N.A., not applicable; Poss., possibly; Prob., probably; and Toler., tolerable.

Variant nomenclature (NM_032539.4)	Individual with NDD			Frequency		*In silico* predictions			Functional effects					Variant interpretation (ACMG/AMP)	
	Ind.	Sex	Inheritance	M	F	PPH2	SIFT	CADD	Domain	Affects ML/DT	Affects I-PTPδ	Affects SF	Affects Res-Syn	Criteria	Class
Potential disease-causing variants in individuals with NDD:															
c.1381G>T, p.E461*	P1	M	de novo	-	-	N.A.	N.A.	35	N.A.	yes	yes	yes	yes	PM2, PS2, PS3, PP3	4_lik_patho
c.221T>C, p.L74S	P7	M	maternal	-	-	Prob. dam.	Delet.	26	Extracell.	yes	yes	yes	yes	PM2, PS3, PP1	4_lik_patho
c.934A>G, p.T312A	P3	M	maternal	-	1	Prob. dam.	Delet.	24.8	Extracell.	no	no	no	yes	PM2, PS3, PP3	4_lik_patho
c.1121C>G, p.P374R	P8	M	de novo	-	-	Prob. dam.	Delet.	26	Extracell.	yes	yes	yes	yes	PM2, PS3, PP1, PP3	4_lik_patho
c.1276C>T, p.R426C	P4	F	de novo	-[b]	-	Prob. dam.	Delet.	28.2	Extracell.	yes	yes	yes	yes	PM2, PS2, PS3, PP3	4_lik_patho
c.628G>A, p.E210K	P1	M	maternal	-	-	Prob. dam.	Delet.	27.2	Extracell.	no	no	no	no	PM2, PP3	3_VUS
c.1531G>A, p.V511M	P5	M	maternal	-	2	Prob. dam.	Delet.	25.2	Extracell.	no	no	no	no	PP3	3_VUS
Variants reported in Decipher:															
c.26G>T, p.S9I	Dc	M	maternal	-	-	Poss. dam.	Delet.	21.7	Signal	no	no	no	N.A.	PM2, PP3	3_VUS
c.44G>A, p.G15E	Dc	M	maternal	-	-	Poss. dam.	Delet.	22.3	Signal	no	no	no	N.A.	PM2, PP3	3_VUS
Other variants identified in individuals with NDD:															
c.601G>A, p.V201I	P1	M	maternal	19	33	Poss. dam.	Delet.	22.8	Extracell.	no	no	no	N.A.	BS2, PP3	2_lik_benign
c.931C>G, p.P311A	P1	M	NA	1	-	Prob. dam.	Delet.	24.6	Extracell.	no	no	no	no	PP3	3_VUS
c.1451G>A, p.R484Q	P1	M	maternal	1	2	Poss. dam.	Delet.	22.7	Extracell.	no	no	no	no	PP3	3_VUS
c.1665G>C, p.E555D	P1	M	NA	-	-	Benign	Delet.	15.6	Extracell.	no	no	no	no	PM2	2_lik_benign
c.2374C>T, p.R792C	P9	M	maternal	2	3	Prob. dam.	Delet.	29.9	Intracell	no	no	no	no	PP3	3_VUS
Other missense variants from GnomAD included in the study:															
c.968G>A, S323N	-	-	-	6	6	Benign	Toler.	16.4	Extracell.	no	no	no	no	BS2, BP4	2_lik_benign
c.1765G>A, V589I	-	-	-	139	128	Benign	Toler.	0.102	Extracell.	no	no	no	no	BS2, BP4	2_lik_benign

Table S2. Stability analysis of LRR1 and LRR2 domain of human SLITRK2 WT and mutant

The free energy of folding (stability) was calculated using FoldX 5.0.

**Model**	***Energy (kcal.mol-1)***
LRR1 (C33-D270) Wild-type	68.49
LRR1 (C33-D270) L74S	75.80
LRR1 (C33-D270) V201I	67.55
LRR1 (C33-D270) E210K	69.77
LRR2 (P341-P579) Wild-type	51.77
LRR2 (P341-P579) P374R	87.92
LRR2 (P341-P579) R426C	52.50
LRR2 (P341-P579) R484Q	52.26
LRR2 (P341-P579) V511M	58.16
LRR2 (P341-P579) E555D	52.24

Table S3. List of all primary and secondary antibodies used at their corresponding dilutions

Antibody	Species	Vendor	Catalog #	Dilution	RRID
SLITRK2	Rabbit	Dr. Jaewon Ko's laboratory	JK177	WB-1:1000	AB_2892626
GM130	Mouse	BD Transduction Laboratories	610822 (clone 35/GM130)	ICC-1:100	AB_398141
TfR	Rabbit	Abcam	ab214039 (clone EPR20584)	ICC-1:100	AB_2904534
VGLUT1	Guinea pig	Millipore	AB5905	ICC-1:200	AB_2301751
GAD67	Mouse	Millipore	MAB5406 (clone 1G10.2)	IHC-1:500<br>ICC-1:100<br>WB-1:1000	AB_2278725
Synaptophysin	Mouse	Sigma-Aldrich	S5768 (clone SVP-38)	ICC-1:1000	AB_477523
PSD-95	Mouse	NeuroMab	75-028 (clone K28/43)	WB-1:1000	AB_2877189
GABA$_A$γ2	Rabbit	Synaptic Systems	224 003	ICC-1:500	AB_2263066
VGAT	Guinea pig	Synaptic Systems	131 004	IHC-1:500,<br>ICC-1:200	AB_887873
HA	Mouse	BioLegend	901501 (clone 16B12)	ICC-1:500,<br>WB-1:1000	AB_2565006
MAP2	Rabbit	Abcam	ab32454	ICC-1:500	AB_776174
MAP2	Mouse	Sigma-Aldrich	M1406 (clone AP-20)	ICC-1:300	AB_477171
TrkB	Rabbit	Cell Signaling	4603 (clone 80E3)	WB-1:1000	AB_2155125
phospho-TrkB	Rabbit	Thermo Fisher	PA5-36695	WB-1:1000	AB_2553666
BiP	Rabbit	Cell Signaling	3177 (clone C50B12)	WB-1:500	AB_2119845
GFP	Goat	Rockland	600-101-215	ICC-1:500	AB_218182
GluN1	Mouse	Millipore	MAB363 (clone 54.1)	WB-1:1000	AB_94946
NR2A	rabbit	Millipore	07-632	WB-1:1000	AB_310837
β-actin	Mouse	Santa Cruz	sc-47778 (clone C4)	WB-1:1000	AB_2714189
NeuN	Mouse	Millipore;	MAB377 (clone A60)	WB-1:1000	AB_2298772
VGLUT1	Rabbit	Dr. Jaewon Ko's laboratory	JK111	IHC-1:500,<br>ICC-1:300	AB_2810945
PSD-95	Rabbit	Dr. Jaewon Ko's laboratory	JK016	IHC-1:500	AB_2722693
Shank	Rabbit	Dr. Eunjoon Kim's laboratory	1172	ICC-1:200	AB_2810261
GluA1	Rabbit	Dr. Eunjoon Kim's laboratory	1193	WB-1:1000	AB_2722772
GluA2	Rabbit	Dr. Eunjoon Kim's laboratory	1195	WB-1:1000	AB_2722773
Cy3-AffiniPure Donkey Anti-Rabbit IgG antibodies	Rabbit	Jackson ImmunoResearch	711-165-152	IHC-1:500 ICC-1:500	AB_2722772
Cy3-AffiniPure Donkey Anti-Mouse IgG antibodies	Mouse	Jackson ImmunoResearch	715-165-150	IHC-1:500 ICC-1:500	AB_2340813
Cy3-Donkey Anti-Human IgG antibodies	Human	Jackson ImmunoResearch	709-165-149	ICC-1:500	AB_2340535
Cy3-Donkey Anti-Guinea Pig IgG antibodies	Guinea Pig	Jackson ImmunoResearch	706-035-148	ICC-1:500	AB_ 2340447
FITC-AffiniPure Donkey Anti-Mouse	Mouse	Jackson ImmunoResearch	715-095-150	ICC-1:150	AB_2340792

IgG antibodies					
FITC-AffiniPure Donkey Anti-Goat IgG antibodies	Goat	Jackson ImmunoResearch	705-095-147	ICC-1:150	AB_2340401
FITC-AffiniPure Donkey Anti-Rabbit IgG antibodies	Rabbit	Jackson ImmunoResearch	711-095-152	ICC-1:150	AB_2315776
Goat Anti-Guinea Pig IgG antibodies	Guinea Pig	Thermo Fisher	A-21450	ICC-1:100	AB_141882
HRP-goat anti-human IgG antibody	Human	Thermo Fisher	62-8420	WB-1:10000	AB_2533962

Abbreviations: ICC, immunocytochemistry; IHC, immunohistochemistry; WB, western blotting

Supplementary Notes

Further details of participants and molecular analyses

Individuals P1 and P2

Clinical data

Individual P1, a 31-year-old Caucasian male, was born at term from healthy non consanguineous parents with mild macrosomia (weight: 4,500 g, height: 51 cm, OFC: 36 cm). He had early hypotonia but walked independently at the age of 13 months. He had a speech delay and his evolution was marked by a moderate ID with learning difficulties for which he attended a special school. He also developed important behavioral troubles including anxiety and aggressiveness that are drug resistant and alter his familial and social relationships. EEG and brain MRI were normal. On clinical examination his weight was 68 kg, and eight 179 cm, his OFC was 60.5 cm. He was able to make short sentences but was not able to read (**Table 1**). We noted mild facial features that included triangular face, downslanting palpebral fissures, downturned corners of the mouth, large ears and horizontal eyebrows. Patient P1's brother, individual P2, was 33-year-old at time of examination. He also had early hypotonia but walked independently at the age of 13 months, had a speech delay with mild ID. He developed major anxiety, was able to work but was very slow in daily activities. He had no seizures but his EEG was normal as well as his brain MRI. On clinical examination his weight was 68 kg, and eight 180 cm, his OFC was 59.5 cm. He was not clearly dysmorphic but we note horizontal eyebrows and mildly large and floppy ears (**Table 1**).

Genetic analysis

Exome sequencing (ES) was done in part of diagnostic investigations (Strasbourg University Hospital, France). Briefly, DNA was captured using Medexome kit (Roche) and paired-end sequenced 2 * 100 bp on HiSeq4000 sequencer (Illumina, Genomeast plateform). Parental DNA were captured and sequenced in pools of six individuaks (Trio-pool exome sequencing). Sequencing data were analyzed using STARK bionformatic pipeline using GATK and

CANOES to detect respectively SNV and CNV variants. Variants were annotated and ranked using Varank[1] and AnnotSV[2]. A hemizygous nonsense variant was identified in *SLITRK2* (NM_032539.4): c.1381G>T, p.E461*, which had a CADD score of 35 and was absent in gnomAD database in hemizygous state (Table 2). Sanger Sequencing was performed using BigDye Terminator kit v3.1 cycle sequencing kits and run on an ABI Prism 3730XL DNA Analyzer (Perkin Elmer Applied Biosystems) and confirmed the presence of the variant in his affected brother as well as its absence in the blood of patients' mother (**Fig. 1a**, **Fig. S1a**, and **Table S1**).

Individual P3

Clinical data

Individual 3 was a 21-year-old Caucasian male who was born at term from healthy non consanguineous parents. The birth parameters were not available. He had surgery at age two months for a craniosynostosis on the sagittal suture. He had other skeletal abnormalities including a severe kyphoscoliosis requiring surgery and short stature (height at 133 cm and weight at 31 kg). He had severe feeding difficulties and currently uses g-tube exclusively. His milestone was on the normal range until he was six, then a neurologic regression was observed. On examination, he presented with severe ID and minimal speech, spasticity on the lower limb requiring botox injections and dystonia. He developed multifocal seizures at age 10, that responded to levetiracetam. He started to have an unsteady gate at 6 years and progressed to walker then wheel chair. He did not have any behavioral issue. He experienced recurrent urinary tract infections. His brain MRI showed severe, symmetrical cerebral and cerebellar volume loss with prominence of the cortical sulci and associated ex vacuo dilation of the ventricles, atrophy of the corpus callosum and brainstem and bilateral hippocampal atrophy with increased FLAIR signal (**Table 1**).

Genetic analysis

ES (Columbia University, New York, United States) identified a hemizygous missense variant in *SLITRK2* (NM_032539.4): c.934A>G, p.T312A, which was predicted to be pathogenic by SIFT, PPH2 and MutationTaster softwares with a CADD score of 24.8, and was absent in gnomAD database in hemizygous state. The variant was

inherited from the mother (**Fig. 1a**, **Fig. S1a**, and **Table S1**).

Individual P4

Clinical data

Individual P4 was a 13 year-old Caucasian female without any familial history. She was born at term with disharmonious IUGR (weight: 2,460 g, height: 46 cm, OFC: 34 cm). She had failure to thrive with microcephaly with severe developmental delay and severe ID. On examination, height was 129 cm (-2.5 SDS), weight was 27.3 kg (-2.5 SDS) and OFC 51 cm (3rd percentile). She had important gastrooesophageal reflux and scoliosis. Speech was absent and she could walk with support with an unsteady gait and lower limbs spasticity. She developed generalized seizures at age 11 months partially resistant to drugs. Neuropsychiatric troubles were also noted and included ASD, aggressiveness with self-injury and mutism. Her brain MRI showed thin corpus callosum, white matter diffuse reduction and leukomalacia. Hypertricosis was noted on the upper limbs and the sacral region. Facial facial included coarse face aspect with broad eyebrows, deep set eyes, bulbous nasal tip, full and thick lips and prognathism (**Table 1**).

Genetic analysis

ES was performed through Italian Telethon Undiagnosed Disease Program in which the patient had been included (Naples, Italy) and identified the heterozygous missense variant in *SLITRK2* (NM_032539.4): c.1276C>T, p.R426C, which was predicted to be pathogenic by SIFT, PPH2 and MutationTaster softwares with a CADD score of 28.2 and was absent in gnomAD database in hemizygous state. The mother did not carry the variant, which arose *de novo* (**Fig. 1a**, **Fig. S1a**, and **Table S1**).

Individual P8

Clinical data

Individual P8 was a 11 years-old male born from an uneventful pregnancy. His maternal uncle had isolated epilepsy. His birth weight was 3,250 g (term 40 WG), and height was 52 cm. He had a mild developmental delay with slight delay in verbal abilities and mild ID. He developed focal seizures at age 8, which were sensible to valproate acid. EEG showed left centrotemporal epileptic discharges. He had no neurological regression nor dystonia but had significative anxiety, became easily frustrated, had executives and interaction difficulties in the context of ADHD. He attended mainstream school with extra help. At age 11, his height was 142.5 cm and his weight was 30 kg. Brain MRI showed unspecific minor white substance punctate changes on the right side around trigonum (**Table 1**).

Genetic analysis

Trio ES (Dianalund, Denmark) was performed as followed: after enrichment of exonic DNA fragments with a SureSelect Human All Exon Kit (Agilent, 50 Mb V5), sequencing was performed on a HiSeq2500 system (Illumina). The genomic regions targeted by the respective enrichment design had an average coverage of > 100 reads and > 97% were covered by =/> 10 reads. An hemizygous missense variant was identified in *SLITRK2* (NM_032539.4): c.1121C>G, p.P374R, predicted to be pathogenic by SIFT, PPH2 and MutationTaster softwares with a CADD score of 26 and was absent in gnomAD database in hemizygous state. Sanger sequencing was performed to confirm the variant, which was inherited from the asymptomatic mother but arose *de novo* in the latter since the maternal grandparents did not carry it (**Fig. 1a**, **Fig. S1a**, and **Table S1**).

Individual P5

Clinical data

Individual P5 was a 12 years-old male without any family history. He was born prematurely at term 25 WG with a weight of 770 g and a height of 33.5 cm. He had a persistent ductus arteriosus in this context. In the first months he had significant feeding difficulties with GOR and failure to thrive leading to a percutaneous endoscopic gastrostomy. He still had short stature and low weight (5th centile at age 7). He was followed for a strabismus and a lumbar scoliosis. Motor and language development were slightly delayed and he had a moderate to severe ID. He had

spastic diplegia and dystonia with an unsteady gait requiring a walker and a wheelchair for support getting around. He was anxious and had signs if ADHD, which was not formerly assessed. He was not clearly dysmorphic. Brain MRI performed at the age of 11 months showed bilateral periventricular leukomalacia, enlarged lateral ventricles and a paucity of the white matter (**Table 1**).

Genetic analysis

Trio ES was performed at the Yale Center for Genome Analysis (YCGA). Genomic DNA was captured using the VCRome capture kit followed by Illumina DNA sequencing. WES data was processed using two independent pipelines at the Yale School of Medicine and Phoenix Children's Hospital. At each site sequence reads were mapped to the reference genome (GRCh37) with BWA-MEM and further processed using GATK Best Practice workflows, which include duplication marking, indel realignment, and base quality recalibration. Single nucleotide variants and small indels were called with GATK HaplotypeCaller and annotated using ANNOVAR, dbSNP (v138), 1000 Genomes (August, 2015), NHLBI Exome Variant Server (EVS), and the Exome Aggregation Consortium v3 (ExAC). Rare deleterious missense variants and LOF variants (stop-gain, stop-loss, frameshift insertions/deletions, canonical splice site, and start-loss) were selected. MetaSVM and Combined Annotation Dependent Deletion (CADD v1.3) algorithms were used to predict deleteriousness of missense variants (MetaSVM-deleterious or CADD ≥ 20). A hemizygous missense variant was identified in *SLITRK2* (NM_032539.4): c.1531G>A p.V511M, predicted to be pathogenic by SIFT, PPH2 and MutationTaster softwares with a CADD score of 25.2 and was absent in gnomAD database in hemizygous state. The variant was inherited from the asymptomatic mother; the maternal grand-parents were not tested (**Fig. 1a**, **Fig. S1a**, and **Table S1**).

Individual P10

Clinical data

Individual P10 is an 11 year-old boy with no familial history of neurodevelopmental disabilities. He was born at full term via C-section to a 29 year-old G1 mother. Apgar score was 8 at both 1 and 5 minutes. He had a pneumothorax

diagnosed shortly after birth due to respiratory symptoms, but was discharged home at 5 days of age. He had feeding difficulties in infancy due to gastroesophageal reflux, and was fussy and "gassy". He had abdominal pain as he got older and was found to have mildly elevated liver enzymes that later normalized. Otherwise he has been healthy and well grown. He achieved all gross motor milestones on time, but he had delays in fine motor skills development and has required therapy services. Language development was normal, but he received therapy for issues with pragmatic speech. He does not have intellectual disabilities, but he has significant neuropsychological problems, including ADHD, anxiety, obsessive compulsive behaviors, tantrums, vocal tics and features of autism (poor socialization, sensitivity to loud sounds, repetitive behaviors). His anxiety has improved since he has begun taking low dose fluoxetine. At 11 years, his height is 144.8 cm and weight is 42 kg. OFC at 8 years was 53.8 cm. He is non-dysmorphic and physical examination is normal. Brain MRI has not been performed (**Table 1**).

Genetic analysis

ES (Washington University School of Medicine, Saint-Louis, United States) identified a hemizygous missense variant in *SLITRK2* (NM_032539.4): c.628G>A, p.E210K, which was predicted to be pathogenic by SIFT, PPH2 and MutationTaster softwares with a CADD score of 27.2 and was absent in gnomAD database in hemizygous state. The variant was inherited from the asymptomatic mother (**Fig. 1a**, **Fig. S1a**, and **Table S1**).

Individual P7

Clinical data

Individual P7 was a 12 years-old boy with no familial history. He was born prematurely at 34 WG with a weight at 2100g. Early hypotonia was noticed followed by a developmental delay, including speech delay. At presented with joint laxity, pedes planovalgi and mild thoracal scoliosis with increased lumber lordosis. At age 9 the height was 146 cm, the weight was 43.2 kg and the OFC was 55.7 cm. He had no epilepsy nor dystonia but had an unsteady gait. He developed a severe ID (IQ 20-36 at age 12 years). He had mild facial dysmorphic features including mild facial asymmetry, slightly broad forehead, full nasal tip, long and smooth philtrum, thin lips and prominent ears

(photographs not available). He was diagnosed with an ASD, important anxiety and hyperactivity. A brain MRI was not done (**Table 1**).

Genetic analysis

ES (Radboud university medical center, Nijmegen, The Netherlands) identified the hemizygous missense variant in *SLITRK*2 (NM_032539.4) c.221T>C, p.L74S, which was predicted to be pathogenic by SIFT, PPH2 and MutationTaster softwares with a CADD score of 26,4 and was absent in gnomAD database in hemizygous state. The variant was inherited from the asymptomatic mother, as well as a hemizygous pathogenic variant in the *ARX* gene (c.1109C>T, p.A370V)[3] (See also **Fig. 1a** and **Table S1**). The maternal grandparents were not available but both variants were absent in the healthy maternal uncle.

Supplementary References

1. Geoffroy, V., et al. VaRank: a simple and powerful tool for ranking genetic variants. *PeerJ* 3, e796 (2015).

2. Geoffroy, V., et al. AnnotSV: an integrated tool for structural variations annotation. *Bioinformatics* 34, 3572-3574 (2018).

3. Thai, M.H.N., et al. Constraint and conservation of paired-type homeodomains predicts the clinical outcome of missense variants of uncertain significance. *Hum Mutat*, 41, 1407–1424. (2020)
