# Supplementary material for: SLITRK2 variants associated with neurodevelopmental disorders impair excitatory synaptic function and cognition in mice
Source: Nat Commun. 2022 Jul 15;13:4112. doi: 10.1038/s41467-022-31566-z (PMC9287327; doi:10.1038/s41467-022-31566-z)
Supplement: Supplementary file 4 — Reporting Summary [file 41467_2022_31566_MOESM4_ESM.pdf]

Corresponding author(s): Ji Won Um
Last updated by author(s): Jun 13, 2022

Reporting Summary

Nature Portfolio wishes to improve the reproducibility of the work that we publish. This form provides structure for consistency and transparency in reporting. For further information on Nature Portfolio policies, see our Editorial Policies and the Editorial Policy Checklist.

Statistics

For all statistical analyses, confirm that the following items are present in the figure legend, table legend, main text, or Methods section.

n/a	Confirmed	
☐	☒	The exact sample size (n) for each experimental group/condition, given as a discrete number and unit of measurement
☐	☒	A statement on whether measurements were taken from distinct samples or whether the same sample was measured repeatedly
☐	☒	The statistical test(s) used AND whether they are one- or two-sided<br>*Only common tests should be described solely by name; describe more complex techniques in the Methods section.*
☒	☐	A description of all covariates tested
☐	☒	A description of any assumptions or corrections, such as tests of normality and adjustment for multiple comparisons
☐	☒	A full description of the statistical parameters including central tendency (e.g. means) or other basic estimates (e.g. regression coefficient) AND variation (e.g. standard deviation) or associated estimates of uncertainty (e.g. confidence intervals)
☐	☒	For null hypothesis testing, the test statistic (e.g. F, t, r) with confidence intervals, effect sizes, degrees of freedom and P value noted<br>*Give P values as exact values whenever suitable.*
☒	☐	For Bayesian analysis, information on the choice of priors and Markov chain Monte Carlo settings
☒	☐	For hierarchical and complex designs, identification of the appropriate level for tests and full reporting of outcomes
☒	☐	Estimates of effect sizes (e.g. Cohen's d, Pearson's r), indicating how they were calculated

Our web collection on statistics for biologists contains articles on many of the points above.

Software and code

Policy information about availability of computer code

Data collection	Zen v2.3 (Zeiss) was used to acquire confocal and two-photon images. Clampex 10/Multiclamp 700B was used to capture electrophysiological traces. Ethovision XT10.5 were used to collect video data for behavioral analysis.
Data analysis	Excel (Microsoft), Prism v7 (Graphpad), ImageJ v1.5a (NIH) and MetaMorph (Molecular Devices) were used to analyze data and images, and to draw graphs. Clampfit 10.8 (Molecular Devices) and OriginPro 8.5 software (OriginLab) were used for electrophysiological analysis.

For manuscripts utilizing custom algorithms or software that are central to the research but not yet described in published literature, software must be made available to editors and reviewers. We strongly encourage code deposition in a community repository (e.g. GitHub). See the Nature Portfolio guidelines for submitting code & software for further information.

Data

Policy information about availability of data

All manuscripts must include a data availability statement. This statement should provide the following information, where applicable:

- Accession codes, unique identifiers, or web links for publicly available datasets
- A description of any restrictions on data availability
- For clinical datasets or third party data, please ensure that the statement adheres to our policy

Data sets presented in this study are included in full wherever possible, including the display of individual data points. All relevant data supporting the findings of this study are available from the corresponding authors upon reasonable request. Biological materials, including mutant mice and custom antibodies generated for this study, will be shared upon request within the limits of respective material transfer agreements for as long as they are available in the laboratory. Source data are provided with this paper.

Field-specific reporting

Please select the one below that is the best fit for your research. If you are not sure, read the appropriate sections before making your selection.

- [x] Life sciences
- [ ] Behavioural & social sciences
- [ ] Ecological, evolutionary & environmental sciences

For a reference copy of the document with all sections, see nature.com/documents/nr-reporting-summary-flat.pdf

Life sciences study design

All studies must disclose on these points even when the disclosure is negative.

Sample size	We did not perform power analysis to predetermine sample sizes, but our sample sizes are similar to those generally employed in the field. (ref. 10, 14, 17, 19, 45).
Data exclusions	No data were excluded from the analysis.
Replication	We used the number of repetitions that are standard in the field for immunocytochemistry, electrophysiology, and mouse behaviors. These values represent the minimum number of repetitions/animals needed to obtain confidence in any experimental outcome and to rule out observations that might be due to animal to animal and/or culture to culture variability. It has been our experience that 5 - 6 repetitions are the minimum required to have any confidence in the observation. Within each replicate there are duplicates and paired negative and positive controls to control for variability from animal to animal or slice to slice within the same day's experiment (duplicates) and across experiments done on different days (paired negative and positive controls). Controlling for variability and guaranteeing reproducibility is the standard necessary for publication of these studies in a peer-reviewed journal. All attempts to replicate the results were successful.
Randomization	Mice were group-housed with same sex siblings and from each group (4 mice per cage) until they were randomly selected to participate in experiments. In addition, mice were identified with specific ear tag number; thus, their identity was hidden until test was ended.<br>For all other experiments, samples were also collected, coded, and analyzed blindly, as indicated in the data analyses part.
Blinding	Analysis was carried out blind, except for experimental conditions that were evident from the image data.

Reporting for specific materials, systems and methods

We require information from authors about some types of materials, experimental systems and methods used in many studies. Here, indicate whether each material, system or method listed is relevant to your study. If you are not sure if a list item applies to your research, read the appropriate section before selecting a response.

Materials & experimental systems

n/a	Involved in the study	
[ ]	[x]	Antibodies
[ ]	[x]	Eukaryotic cell lines
[x]	[ ]	Palaeontology and archaeology
[ ]	[x]	Animals and other organisms
[ ]	[x]	Human research participants
[x]	[ ]	Clinical data
[x]	[ ]	Dual use research of concern

Methods

n/a	Involved in the study	
[x]	[ ]	ChIP-seq
[x]	[ ]	Flow cytometry
[x]	[ ]	MRI-based neuroimaging

Antibodies

Antibodies used	Primary antibodies: customized antibodies (JK177, JK016, JK111, 1172, 1193, 1195), mouse monoclonal anti-GM130 (clone 35/GM130; BD Transduction Laboratories; Cat# 610822; RRID: AB_398141), rabbit monoclonal anti-TfR (clone EPR20584; Abcam; Cat# ab214039; RRID: AB_2904534), guinea pig polyclonal anti-VGLUT1 (Millipore; Cat# AB5905; RRID: AB_2301751), mouse monoclonal anti-GAD67 (clone 1G10.2; Millipore; Cat# MAB5406; RRID: AB_2278725), mouse monoclonal anti-Synaptophysin (clone SVP-38; Sigma-Aldrich; Cat# S5768; RRID: AB_477523), mouse monoclonal anti-PSD-95 (clone K28/43; NeuroMab; Cat# 75-028; RRID: AB_2877189), rabbit polyclonal anti-GABAAγ2 (Synaptic Systems; Cat# 224 003; RRID: AB_2263066), guinea pig polyclonal anti-VGAT (Synaptic Systems; Cat# 131 004; RRID: AB_887873), mouse monoclonal anti-HA (clone 16B12; BioLegend; Cat# 901501; RRID: AB_2565006), mouse monoclonal anti-MAP2 (clone AP-20; Sigma-Aldrich; Cat# M1406; ICC-1:300; AB_477171), rabbit polyclonal anti-MAP2 (Abcam; Cat# ab32454; AB_776174),rabbit monoclonal anti-TrkB (clone 80E3; Cell Signaling; Cat# 4603; RRID: AB_2155125), rabbit monoclonal anti-phospho-TrkB (Thermo Fisher; Cat# PA5-36695; RRID: AB_2553666), rabbit monoclonal anti-BiP (clone C50B12; Cell Signaling; Cat# 3177; RRID: AB_2119845), goat polyclonal anti-GFP (Rockland; Cat# 600-101-215; RRID: AB_218182), mouse monoclonal anti-GluN1 (clone 54.1; Millipore; Cat# MAB363; RRID: AB_94946), rabbit polyclonal anti-NR2A (Millipore; Cat# 07-632; RRID: AB_310837), mouse monoclonal anti-β-actin (clone C4; Santa Cruz; Cat# sc-47778; RRID: AB_2714189), mouse monoclonal anti-NeuN (clone A60; Millipore; Cat# MAB377; RRID: AB_2298772)<br><br>Secondary antibodies: HRP-goat anti-human IgG crossed-adsorbed secondary antibody (Thermo Fisher; Cat# 62-8420; RRID:

	AB_2533962), Cy3-AffiniPure donkey Anti-rabbit IgG antibodies (Jackson ImmunoResearch; Cat# 711-165-152; RRID: AB_2307443), Cy3-AffiniPure donkey anti-mouse IgG antibodies (Jackson ImmunoResearch; Cat# 715-165-150; RRID: AB_2340813), Cy3-donkey anti-human IgG antibodies (Jackson ImmunoResearch; Cat# 709-165-149; RRID: AB_2340535), Cy3-donkey anti-guinea pig IgG antibodies (Jackson ImmunoResearch; Cat# 706-035-148; RRID: AB_ 2340447), FITC-AffiniPure donkey anti-mouse IgG antibodies (Jackson ImmunoResearch; Cat# 715-095-150; RRID: AB_2340792), FITC-AffiniPure donkey anti-goat IgG antibodies (Jackson ImmunoResearch; Cat# 705-095-147; RRID: AB_2340401), FITC-AffiniPure Donkey anti-rabbit IgG antibodies (Jackson ImmunoResearch; Cat# 711-095-152; RRID: AB_2315776), goat anti-guinea pig IgG antibodies (Thermo Fisher; Cat# A-21450; RRID: AB_141882).
Validation	JK177 antibodies were validated by performing immunoblotting using mouse lysates of each KO mouse (shown in Figure 5l). The other customized antibodies (JK016, JK111, 1172, 1193 and 1195) have been validated in previous papers (ref. 17, 47, 51). All commercial antibodies were validated by vendor, as indicated on their websites.

Eukaryotic cell lines

Policy information about cell lines

Cell line source(s)	HEK293T cells were purchased from ATCC.
Authentication	None
Mycoplasma contamination	After receiving a cell line into the laboratory and/or after > 10 passages, mycoplasma testings were performed using Universal Mycoplasma Detection Kit (purchased from ATCC). Cell lines were tested negative for mycoplasma contamination.
Commonly misidentified lines (See ICLAC register)	None of the cell lines used is listed as commonly misidentified.

Animals and other organisms

Policy information about studies involving animals; ARRIVE guidelines recommended for reporting animal research

Laboratory animals	A Nestin-Cre (003771) line in a C57BL/6N genetic background, generated by crossing the original Cre-driver lines purchased from the Jackson Laboratory with C57BL/6J for more than five generations, was obtained from Dr. Albert Chen (DUKE-NUS, Singapore). Slitrk2 floxed mice were generated in collaboration with Biocytogen Co. (China). All experimental procedures were performed on male mice. Mice were kept on a 12:12-h light/dark cycle (lights on at 7:00 am), and received water and food ad libitum at room temperature and 40-60% humidity. All studies used littermate male mice with the same age. 6-10 week-old male mice were used for all immunohistochemical and behavioral tests.
Wild animals	No wild animals were involved in this study.
Field-collected samples	This study did not involve field-collected samples.
Ethics oversight	All procedures were conducted in accordance with the animal care standards outlined in the Guide for the Care and Use of Experimental Animals and were approved by Daegu Gyeongbuk Institute of Science and Technology (DGIST) Administrative Panel on Laboratory Animal Care (DGIST-IACUC-19052109-00).

Note that full information on the approval of the study protocol must also be provided in the manuscript.

Human research participants

Policy information about studies involving human research participants

Population characteristics	The participants of the study, seven males -including sibling- and one female (P1-5, P7-8, P10), are aged 8 years to 33 years. They present with neurodevelopmental disorders (NDDs) including intellectual disability of variable severity, speech impairment, unsteady gait, possible seizures and neuropsychiatric manifestations with major anxiety and autism spectrum disorder. They all were followed by a referring clinical geneticist and underwent whole exome sequencing (WES) in different molecular laboratories in order to identify the molecular cause of their conditions, after negative standard genetic investigations and malformation screening.
Recruitment	All patients were referred to a clinical genetics department in the context of their diagnostic workup for ID. After obtaining of families' written informed consent for diagnostic and research genetics analyses during a genetics consultation, trio exome sequencing was performed in each patient. WES was used in a diagnostic approach in individuals P1 and P2 (Montpellier, France) leading to the identification of a rare de novo likely pathogenic nonsense variation in the X-linked SLITRK2 gene (E461X). An international data sharing was initiated to find other patients with very rare SLITRK2 variants. This allowed us to identify six additional patients with such NDDs who carried de novo (P3, P4) or maternally inherited (P5, P7, P8 (de novo in the mother), P10) nonsynonymous SLITRK2 missense variants predicted to be damaging (T312A, R426C, P374R, L74S, V511M, E210K). The patients were recruited in the following hospitals: individuals P1 and P2 (Strasbourg University Hospital, France); P3 (Columbia University, US); P4 (Federico II University Hospital, italy); P8 (Danish Epilepsy Center, Denmark); P5 (The University of Adelaide, Australia); P10 (Washington University School of Medicine, US); P7 (Radboud university medical center, the Netherlands). There were no self-selection biases that may be present and how these are likely to impact results.
Ethics oversight	Individuals were referred by clinical geneticists for genetic testing as part of routine clinical care. All patients enrolled and/or their legal representative signed informed consent for research use and authorization for publication. All the institutions received local institutional review board (IRB) approval to use these data in research. The main IRB approval was obtained

from the Ethics Committee of the Strasbourg University Hospital (CCPPRB).

Note that full information on the approval of the study protocol must also be provided in the manuscript.
