# Supplementary figures and images for: SLITRK2 variants associated with neurodevelopmental disorders impair excitatory synaptic function and cognition in mice
Source: Nat Commun. 2022 Jul 15;13:4112. doi: 10.1038/s41467-022-31566-z (PMC9287327; doi:10.1038/s41467-022-31566-z)

**Figure 3b**

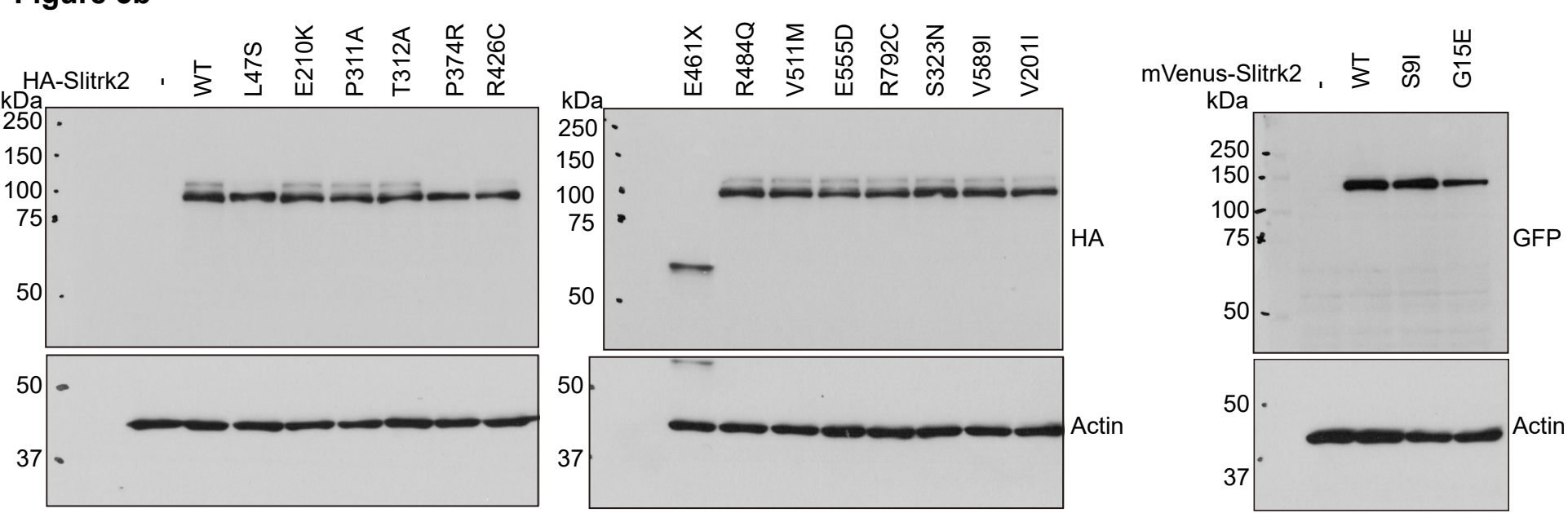

**Figure 5i**

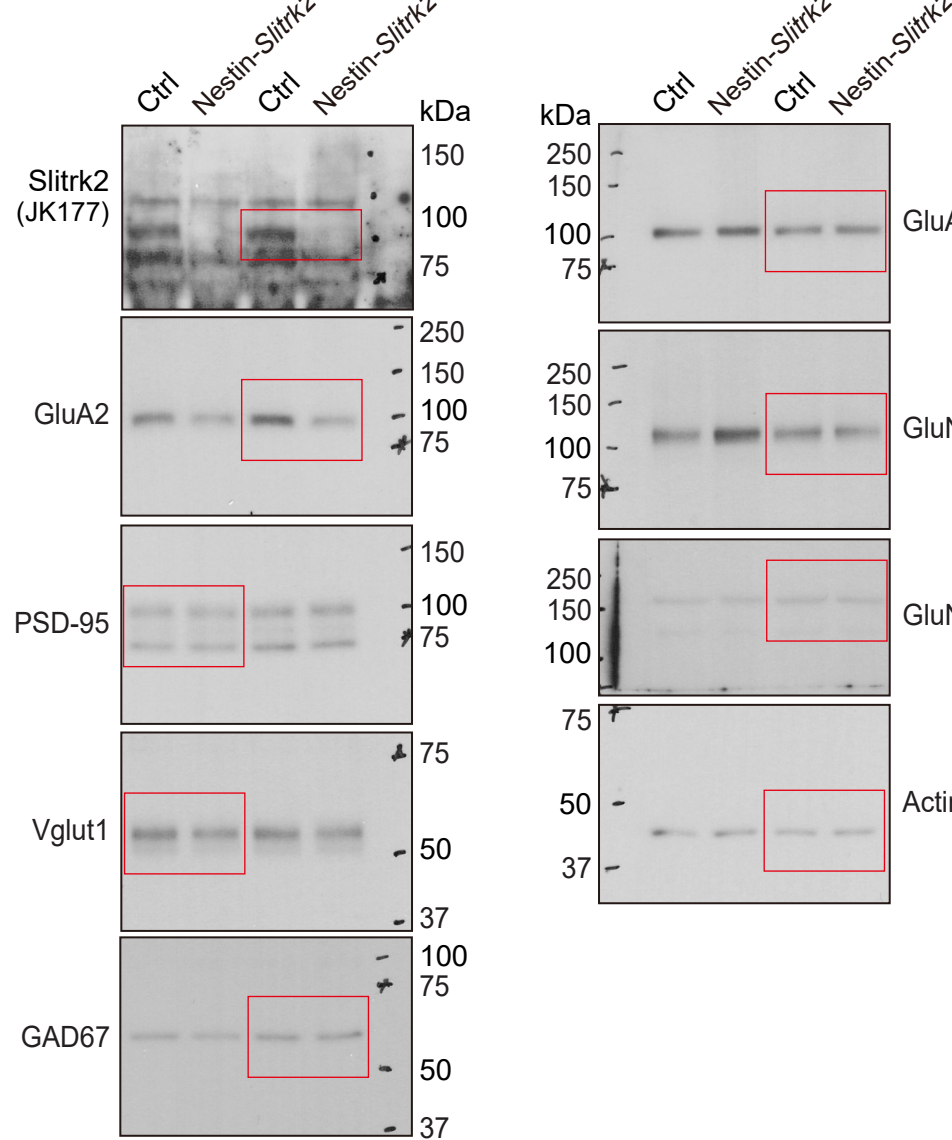

**Figure 7a**

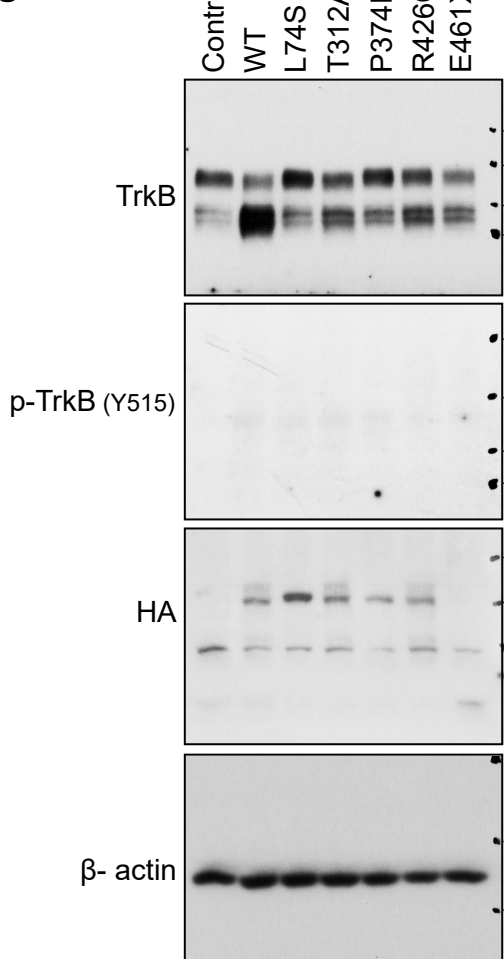

**Figure 7c**

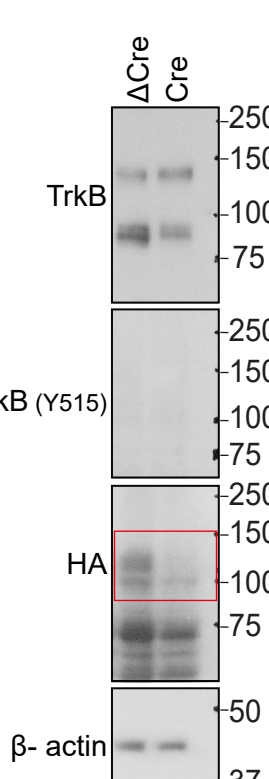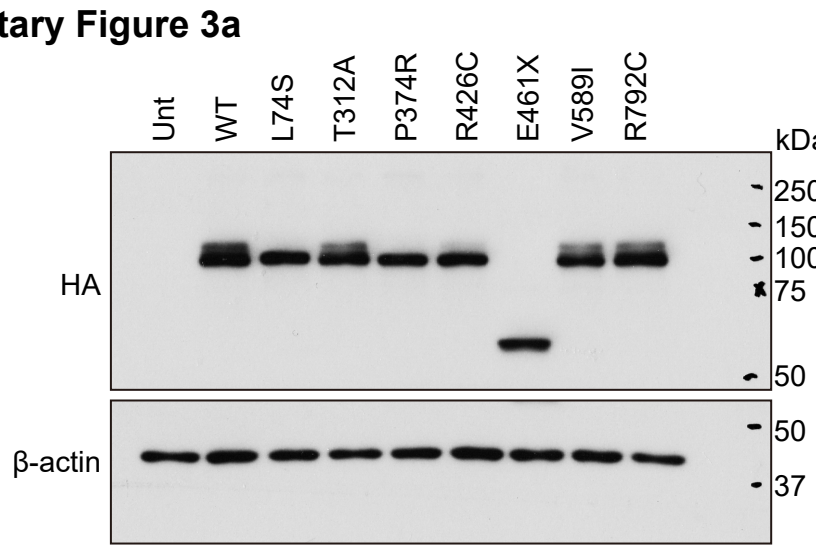

## Conclusion

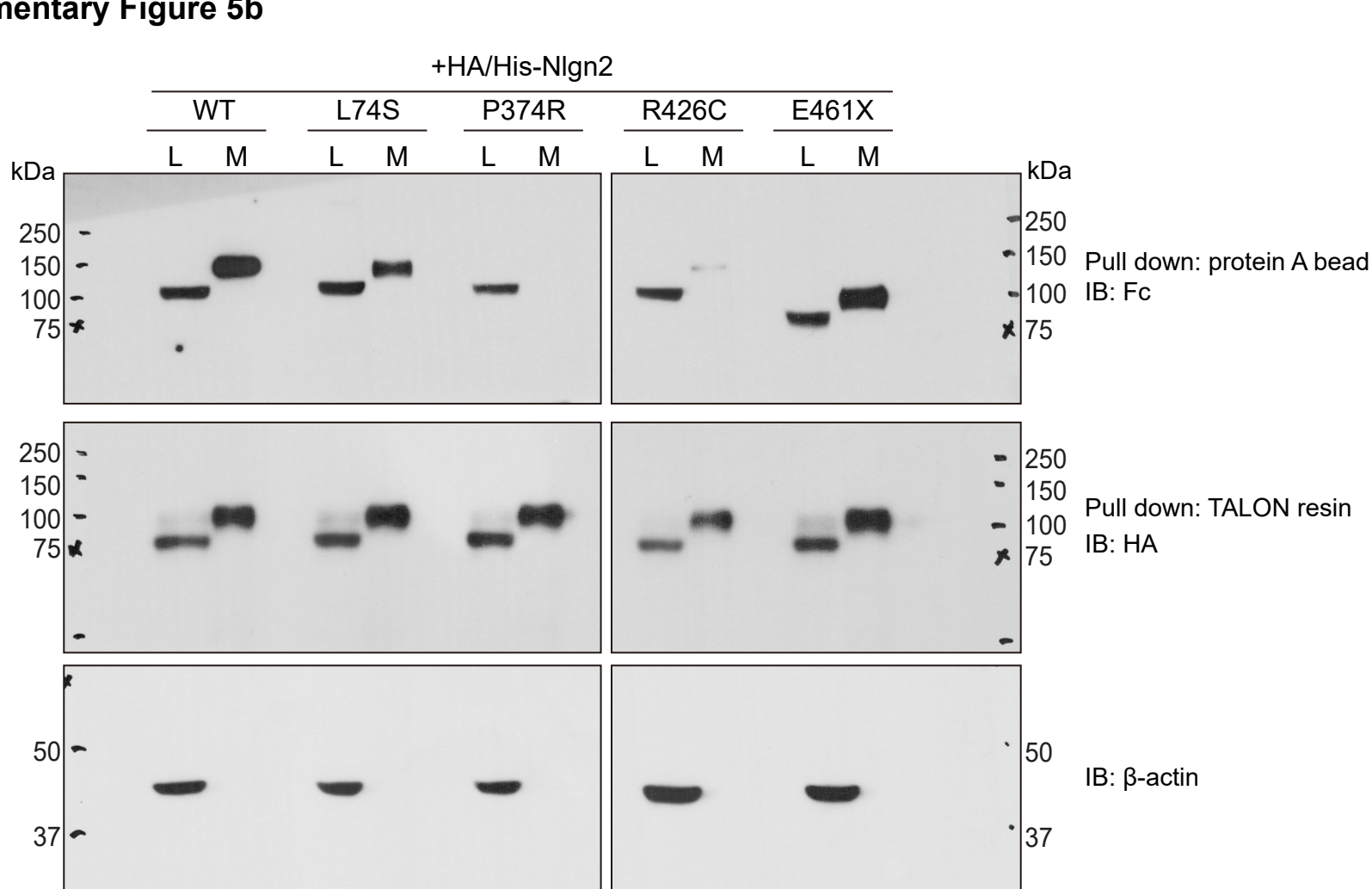

Supplementary Figure 6a

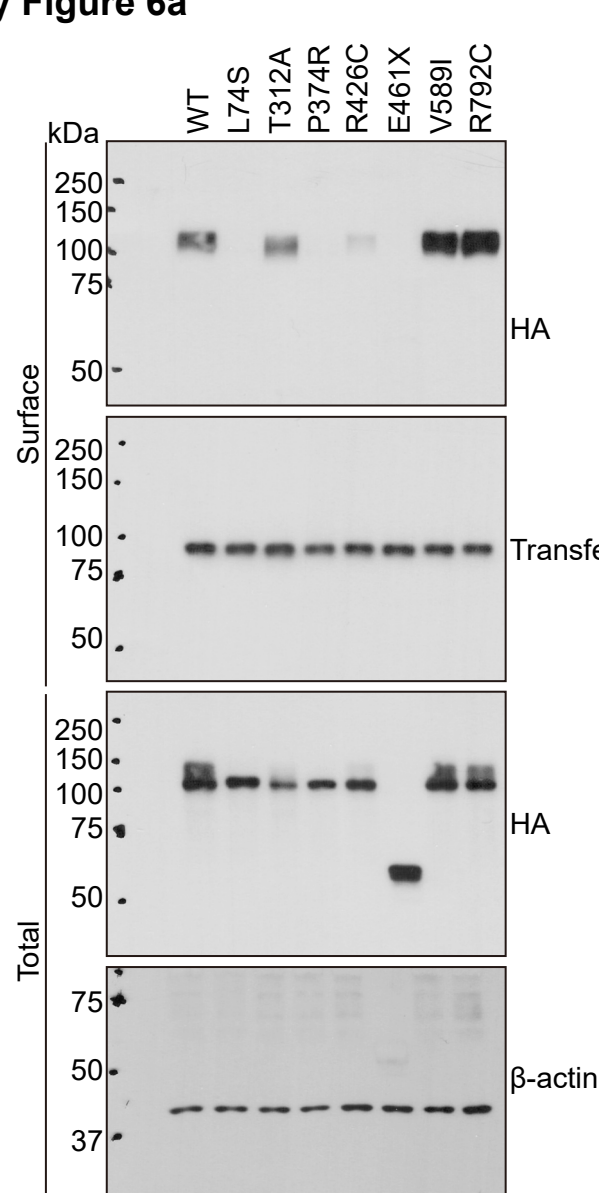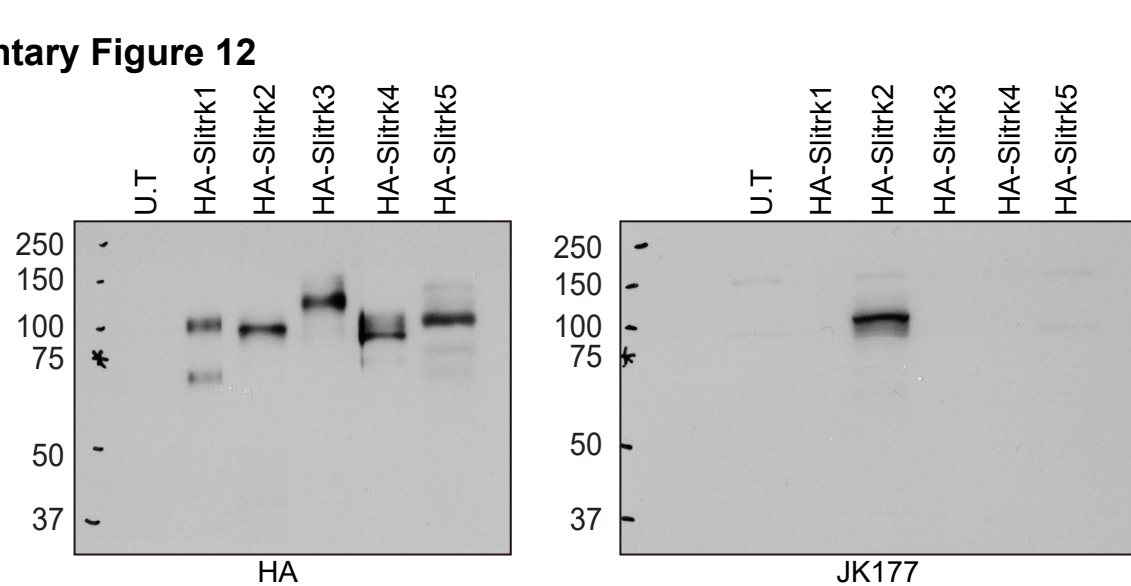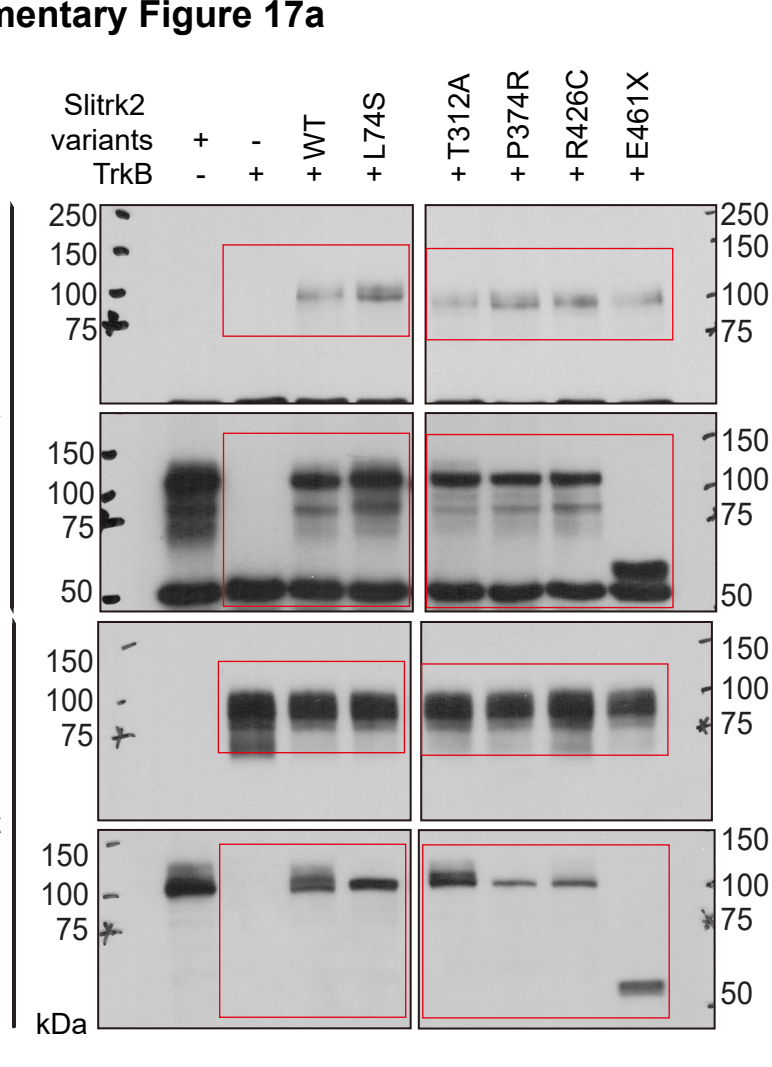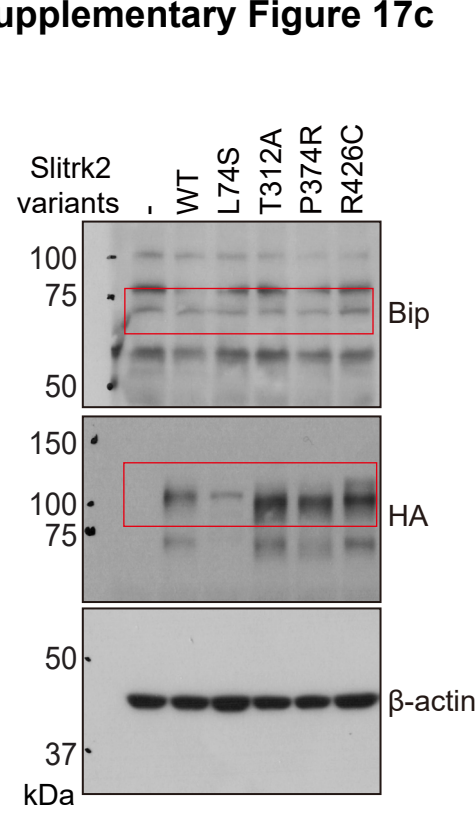

### Supplementary Figure 20

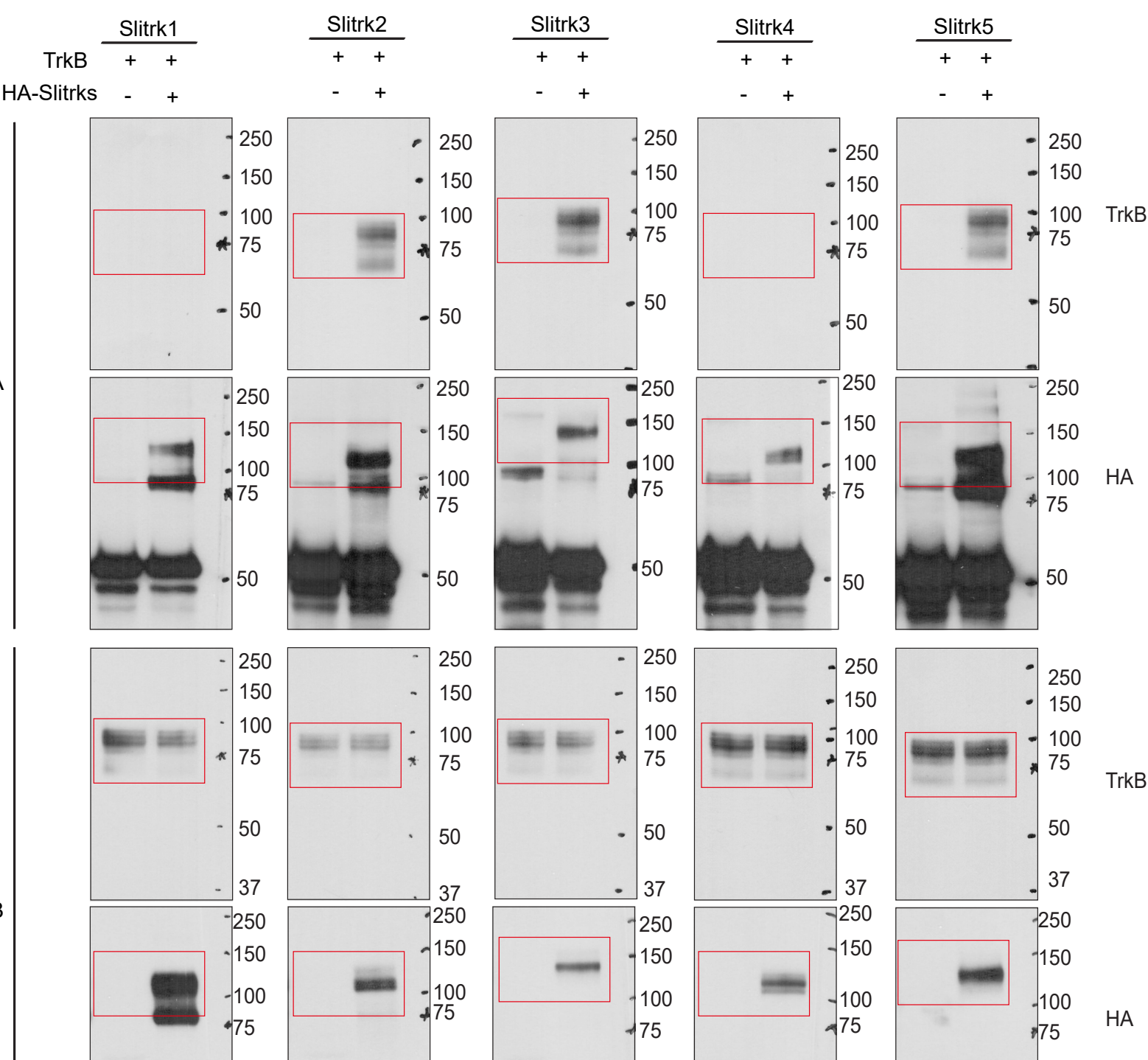

Supplement: Supplementary file 5 — Source Data [file 41467_2022_31566_MOESM5_ESM.zip › Source Data_uncropped immunoblot images.pdf]
